# Co-Processing Agricultural Residues and Wet Organic Waste Can Produce Lower-Cost Carbon-Negative Fuels and Bioplastics

**DOI:** 10.1021/acs.est.2c06674

**Published:** 2023-02-07

**Authors:** Yan Wang, Nawa R. Baral, Minliang Yang, Corinne D. Scown

**Affiliations:** † Energy & Biosciences Institute, University of California, Berkeley, Berkeley, California 94720, United States; ‡ Life-Cycle, Economics, and Agronomy Division, Joint BioEnergy Institute, Emeryville, California 94608, United States; § Biological Systems and Engineering Division, Lawrence Berkeley National Laboratory, Berkeley, California 94720, United States; ∥ Energy Analysis and Environmental Impacts Division, Lawrence Berkeley National Laboratory, Berkeley, California 94720, United States

**Keywords:** bioeconomy, integrated biorefinery, poly(3-hydroxybutyrate), single-cell protein, biogas upgrading, techno-economic analysis, life-cycle assessment, manure management, greenhouse gas emissions

## Abstract

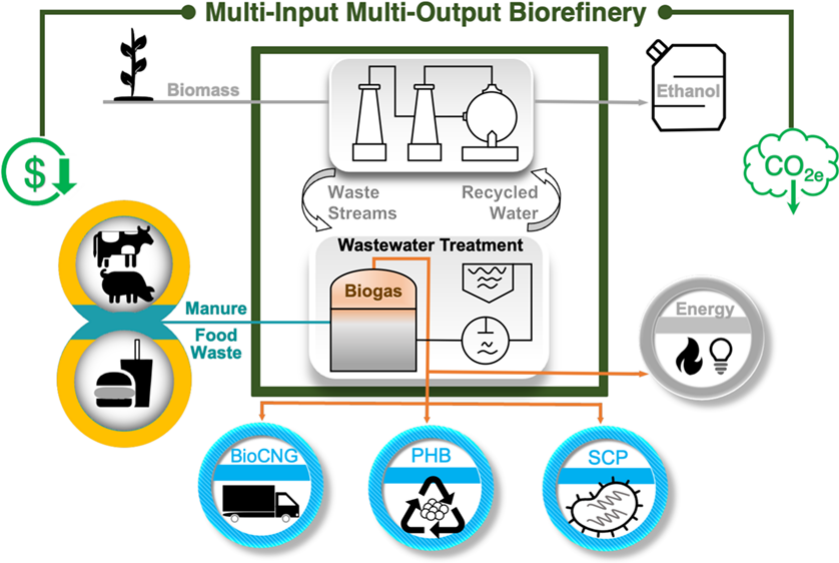

Scalable, low-cost biofuel and biochemical production can accelerate progress on the path to a more circular carbon economy and reduced dependence on crude oil. Rather than producing a single fuel product, lignocellulosic biorefineries have the potential to serve as hubs for the production of fuels, production of petrochemical replacements, and treatment of high-moisture organic waste. A detailed techno-economic analysis and life-cycle greenhouse gas assessment are developed to explore the cost and emission impacts of integrated corn stover-to-ethanol biorefineries that incorporate both codigestion of organic wastes and different strategies for utilizing biogas, including onsite energy generation, upgrading to bio-compressed natural gas (bioCNG), conversion to poly(3-hydroxybutyrate) (PHB) bioplastic, and conversion to single-cell protein (SCP). We find that codigesting manure or a combination of manure and food waste alongside process wastewater can reduce the biorefinery’s total costs per metric ton of CO_2_ equivalent mitigated by half or more. Upgrading biogas to bioCNG is the most cost-effective climate mitigation strategy, while upgrading biogas to PHB or SCP is competitive with combusting biogas onsite.

## Abstract 2

Co-processing wet organic waste and new uses for biogas utilization can broaden the role of lignocellulosic biorefineries and lower the cost of climate change mitigation.

## Introduction

Biological carbon sources have three critical roles to play in reaching global climate change mitigation goals: providing energy-dense fuels for difficult-to-electrify transportation modes, enabling net carbon-negative technologies at lower costs than what is achievable with direct air capture, and replacing petrochemicals with bio-based alternatives.^1,2^ However, the bioeconomy has fallen short of achieving these goals to date. The Energy Independence and Security Act of 2007 set a U.S. cellulosic fuel production target of 16 billion gallons by 2022, but this goal was revised to just 0.63 billion gallons in the latest final volume requirements.^3,4^ This shortfall stems from a variety of factors, including blend wall limitations for ethanol, fluctuating crude oil prices, and challenging economics for the conversion of lignocellulosic material to advanced liquid fuels.^5,6^ The narrow aim of producing a single liquid biofuel as cheaply as possible overlooks the range of services biorefineries can provide. Commercial-scale cellulosic biorefineries have the potential to play a multifaceted role in the future carbon economy by serving as both fuel production and waste treatment infrastructure, ultimately producing multiple fuel and non-fuel products.

Of the U.S. anthropogenic methane emissions, landfilled organic wastes contribute an estimated 20% and manure management is responsible for an additional 9%.^7^ Concentrated animal feeding operations in the U.S. produce approximately 300 million metric tons of waste per year and result in the release of excess nutrients to the environment, causing human health and ecological damage.^8,9^ Much of the recoverable dairy, beef, and swine manure is located in close proximity to current U.S. biorefineries and likely future locations.^10,11^ For example, more than 80% of the total organic waste available in Iowa is manure.^11^ While more densely populated regions have municipal wastewater and other organic waste processing infrastructure that can be leveraged to treat a portion of this waste,^12^ rural communities are less likely to have such centralized infrastructure in place. Lignocellulosic biorefineries have the potential to share the costs and benefits of anaerobic digestion (AD) infrastructure in rural communities, thus mitigating methane emissions and enabling the use of otherwise stranded resources.

In a typical lignocellulosic ethanol biorefinery design, AD is incorporated as part of the wastewater treatment (WWT) section to treat high-biological-oxygen-demand waste streams and produce biogas for onsite energy generation.^13^ The onsite AD facility is an often-overlooked component of most advanced biorefinery designs, yet it has the potential to codigest a range of locally generated organic wastes, such as livestock manure and food processing waste, which are abundant in agricultural areas.^11^ Previous experimental studies and current industry practices have demonstrated the feasibility of codigesting lignocellulosic residues and organic wastes.^12,14,15^ Taking advantage of codigestion, a pragmatic method that overcomes the challenges related to substrate properties and system optimization in a single-substrate AD process, can increase biogas production by supplementing the waste streams of ethanol production with manure and food waste.^12^ Codigestion also presents the opportunity to earn tipping fees as a revenue stream ($42–68 per wet metric ton of waste in the Midwestern U.S.^16^) for accepting organic wastes that would otherwise be landfilled or treated at other private facilities. In addition to the economic advantages, diverting waste from landfills, manure lagoons, and other storage and treatment alternatives can avoid major sources of fugitive methane emissions.

Codigestion of organic wastes alongside biorefinery process wastewater will boost biogas production, raising the question of how this additional biogas can be used. Raw (untreated) biogas from the AD facility at a typical corn stover-to-ethanol biorefinery design is combusted in an onsite combined heat and power (CHP) unit.^13^ Because of low feed-in tariffs for electricity generated from biogas (e.g., $60/MWh without policy support in a recently published case study^17^), there is little to no incentive to maximize the biogas yield as revenue contributions from power sales are minimal.^18^ Future shifts toward renewable electricity will further diminish the benefits of generating power onsite (and credits associated with excess power export to the grid). Cleaning and upgrading biogas to bio-compressed natural gas (bioCNG, also referred to as renewable natural gas) is an attractive alternative to combusting raw biogas onsite. The attractiveness of bioCNG is evidenced by the rapid growth in production; there has been a 24% increase in the dedicated bioCNG production capacity since 2020, totaling 230 operational projects and 188 more either planned or under construction.^19^ BioCNG is eligible for policy incentives aimed at low-carbon fuels as it can be used as a cleaner, renewable alternative to diesel fuel in heavy-duty trucks or directly injected into natural gas pipelines.

An alternative to upgrading biogas is the direct biological conversion of raw biogas to products using methane-oxidizing bacteria (*methanotrophs*). Two well-studied bioproducts that are produced naturally by *methanotrophs* are polyhydroxyalkanoates (PHAs) and single-cell protein (SCP), both of which are being scaled up for commercial production.^20−22^ PHAs can be viable alternatives to petroleum-based polymers (e.g., polypropylene and polyethylene) in some applications, comprising a group of biobased and biodegradable polymers, of which the most abundant and well-studied variety is poly(3-hydroxybutyrate) (PHB).^20^ PHB is mainly used in the packaging industry, although it also has expanded applications in various areas such as medicine, agriculture, and nanocomposites.^23^ High production costs are a major barrier to increasing PHB’s market share (about three to four times more expensive than petroleum-based plastics),^24^ yet growing desire to mitigate plastic waste has stimulated the shift to more compostable plastics, including PHB. Efforts to improve production efficiencies are also bringing the production cost for PHB down.^25,26^ SCP refers to the protein derived from microbial cells and can be a viable alternative to PHB production. SCP has been used for livestock feed, particularly in the aquaculture sector that resembles fish meal in terms of amino acid composition and nutritional quality.^21,22^

**Figure 1:**
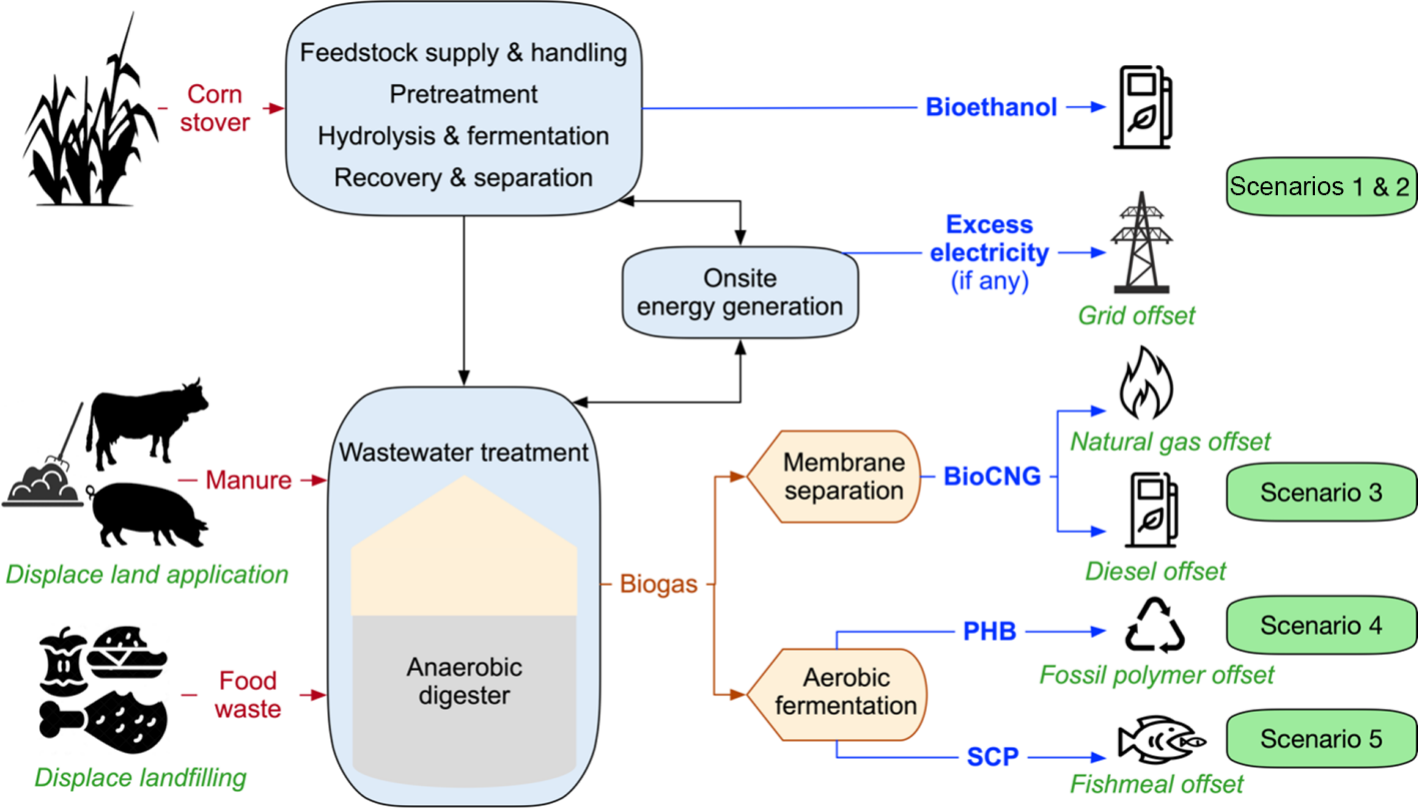
Cradle-to-gate system boundary for TEA and LCA. The environmental credits applicable to this multi-input multi-output biorefinery are indicated by the text in green. CNG: compressed natural gas, SCP: single-cell protein, PHB: poly(3-hydroxybutyrate). Additional details for each scenario are available in Figure 2.

Multiple studies have highlighted the advantages of biorefineries that output a suite of products rather than a single fuel.^27,28^ Some articles have explored the possibility of using mixed feedstocks, although most focus on blending different types of biomass for use in a single conversion process.^29,30^ However, this is the first study that details an approach to designing integrated lignocellulosic biorefineries as hubs for producing liquid fuels, processing organic wastes, and utilizing raw biogas for higher-value fuels and products. Beginning with a corn stover-to-ethanol biorefinery, where biogas is combusted onsite, additional scenarios are developed that incorporate both codigestion of organic wastes and different biogas utilization routes to bioCNG, PHB, and SCP (Figure 1). Each scenario is assessed through detailed techno-economic analysis (TEA) and life-cycle assessment (LCA). Specifically, we present cradle-to-gate results for each biorefinery design in two key metrics: minimum ethanol selling price (MESP), referring to the price at which ethanol must be sold to achieve a zero net present value after including a predefined internal rate of return, and life-cycle greenhouse gas (GHG) emissions. The two metrics are then combined to evaluate the cost of carbon mitigation for each design.

## Methods

With corn stover as a representative feedstock, we first simulated a baseline lignocellulosic biorefinery that produces bioethanol. The process design for this corn stover-to-ethanol biorefinery is grounded in a widely used study by the National Renewable Energy Laboratory (NREL),^13^ with the exception of the pretreatment process and AD section of the facility. The original NREL study used a dilute acid (DA) pretreatment process, whereas this study relies on a newer deacetylation and mechanical refining (DMR) process. Our baseline biorefinery combusts lignin, unconverted cellulose/hemicellulose, biogas, and biomass sludge in a CHP unit to generate steam and electricity. Furthermore, we compared results from the baseline biorefinery against integrated biorefineries that incorporate codigestion of organic wastes and different biogas utilization routes to bioCNG, PHB, and SCP. We developed mass and energy balances for each biorefinery configuration and assessed the impacts on the MESP, life-cycle GHG emissions, and cost of carbon mitigation. Unless otherwise stated, metric ton (t) is the standard unit of mass in this study.

### Biorefinery Scenarios

**Figure 2:**
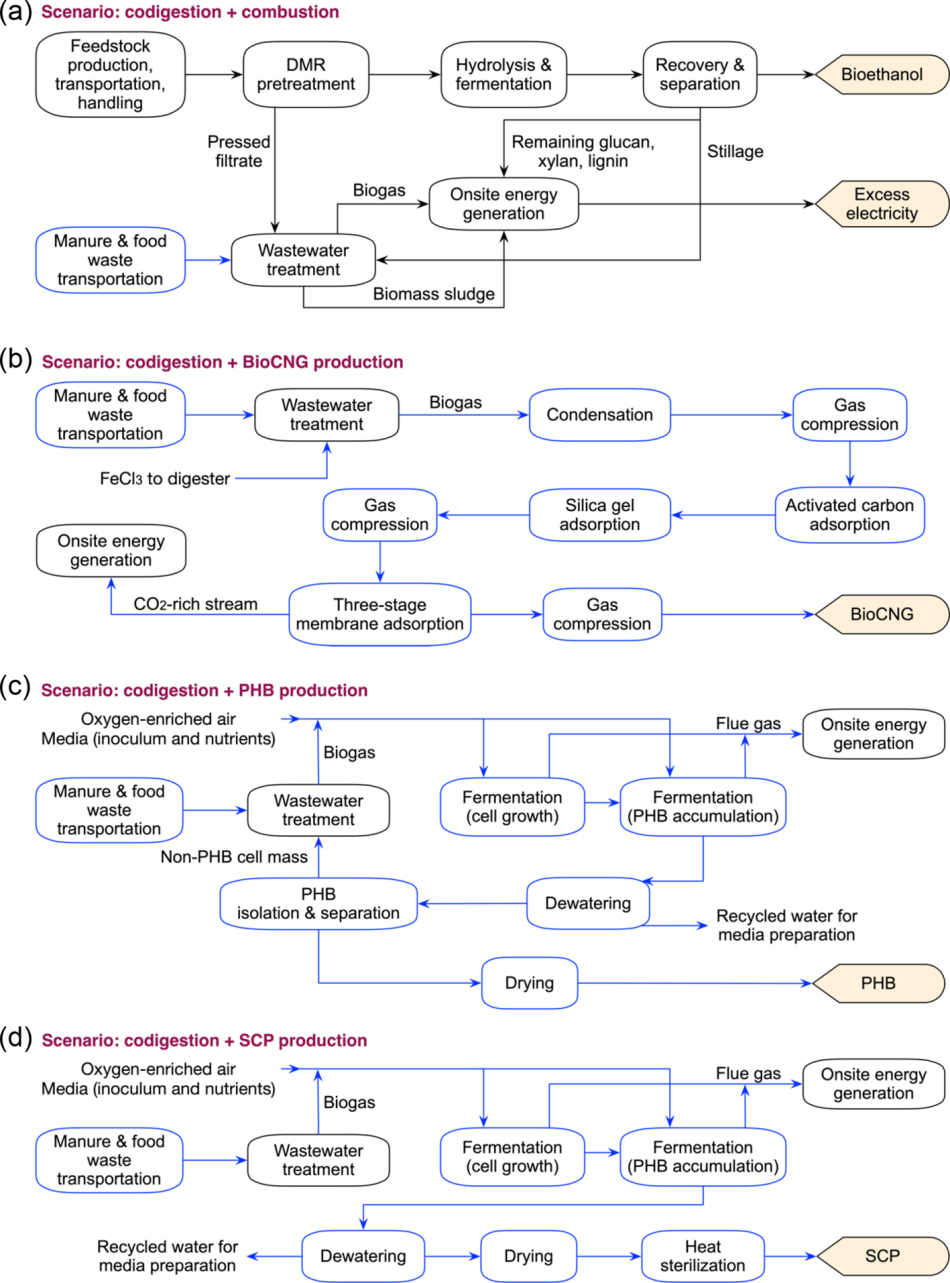
Process flow diagrams for four scenarios, including (a) corn stover-to-ethanol biorefinery incorporating codigestion of organic wastes and an expanded biorefinery further incorporating (b) bioCNG, (c) PHB, and (d) SCP production, respectively. DMR: deacetylation and mechanical refining, CNG: compressed natural gas, SCP: single-cell protein, PHB: poly(3-hydroxybutyrate).

We evaluated five scenarios, each with different WWT and biogas utilization configurations (Figure 2): scenario 1 (S1, Figure 2a) is the baseline corn stover-to-ethanol biorefinery where biogas is combusted onsite to generate heat and electricity. Scenario 2 (S2, Figure 2a) incorporates codigestion of organic wastes (i.e., livestock manure and food waste) with biogas combusted onsite. Scenarios 3–5 (S3–S5, Figure 2b–d) include codigestion of organic wastes and biogas utilization to bioCNG, PHB, and SCP, respectively.

### Corn Stover Production and Logistics

The biorefinery location has an impact on both the economics and life-cycle GHG footprint because the availability of feedstock and regional electricity grid mix will vary. We assume that the location of a biorefinery is in the Corn Belt region of the Midwestern U.S. The farm-to-biorefinery supply radius is assumed to be 50 miles (80 km) with the biorefinery located at the center of a circular feedstock collection area. The composition and supply logistics cost of corn stover are shown in the Supporting Information (SI), Table S1.

### Conversion of Biomass to Bioethanol

Consistent with the NREL study,^13^ the bioethanol production is divided into six process areas (Figures 1 and 2a): (1) feedstock handling, (2) pretreatment, (3) enzymatic hydrolysis and fermentation, (4) ethanol recovery and separation, (5) WWT, and (6) onsite energy generation. A feedstock handling process is required prior to pretreatment including truck unloading, belt conveying, milling, and short-term storage. We updated the pretreatment design in this study. DMR was selected due to its simpler process design, lower chemical and steam usage, higher solid loading, and particularly negligible fermentation inhibitor production compared to other pretreatment methods such as DA, ionic liquid (IL), and ammonia fiber expansion (AFEX) processes.^31^ Furanic and phenolic compounds formed during pretreatment are typical lignocellulose-derived inhibitors to microbes that negatively affect ethanol and methane production.^32^ Both furans and phenolics are present in DA and AFEX processes,^33^ while phenolics are also observed for IL pretreatment.^34^ In contrast, the mild alkaline pretreatment in DMR is potentially advantageous in generating negligible inhibitors.^35^ Unlike biorefineries that use chemical pretreatment methods (e.g., DA or IL),^13,36,37^ in which onsite electricity production exceeds onsite needs for a baseline bioethanol facility, DMR relies on an electricity-intensive mechanical refining process.^31^ For this reason, the baseline biorefinery (S1) can only generate 65% of its power needs. The process details are described in the SI, and the operating conditions for all process areas are compiled in Table S1.

### Organic Waste Availability for Codigestion

Particularly because manure and food waste are high in moisture (80–90% water by mass), local availability of sufficient resources will be important to prevent prohibitively high truck transportation costs and emissions. We estimated the quantities of livestock manure and food waste for codigestion using data sourced from the U.S. Billion-Ton Report.^10^ In the Corn Belt region, hog and cattle are the primary contributors of manure.^10^ The composition of manures and food waste is summarized in Table S2. The average amounts within a farm-to-biorefinery distance of 80 km in the Corn Belt region are approximately 4600, 1100, and 400 t per day on a wet basis, respectively. These daily amounts of organic wastes are assumed to be transported from their generation sites to the AD facility in the biorefinery (Figure 2a). An important caveat is that many of the potential locations in the Corn Belt do not implement source separation for organic waste, and municipal organic waste streams are often too contaminated to be used directly in a wet AD system without substantial pretreatment. Commercial or industrial sources, such as food processors, grocery stores, and restaurants, may produce more readily processable food waste streams. Additionally, a challenge for codigestion facilities is to ensure that all feedstocks are timely scheduled to deliver during weekdays and to reduce waste hauling time during weekends.^12^ While organic wastes are abundant and close to biorefineries, onsite storage can be used to mitigate this problem.

### Prediction of Biogas Production from Anaerobic Codigestion

Biogas yield and composition are the result of complex dynamics among a consortium of microbes, whose growth is inhibited/enhanced by a range of environmental factors including temperature, pH, and carbon-to-nitrogen ratio. We predicted the biogas yield and composition by combining (i) the theoretical reaction equation of biogas production on the basis of elemental compositions of organic components and (ii) the empirical methane yields for the waste streams flowing to the AD facility, including the pressed filtrate after DMR pretreatment, the stillage after ethanol recovery, manures, and food waste. The latter (ii) determines the conversion rate of the reaction equation (i) for each organic component in the WWT influent. The details for (i) and (ii) can be found in the SI (Table S2 and Figure S1).

### Biogas-to-BioCNG Process

Raw biogas from the AD section of the facility consists mainly of CH_4_ (50–75%) and CO_2_ (25–50%), while trace amounts of other components (e.g., H_2_O, H_2_S, and NH_3_) can be present. If this biogas is routed for the production of bioCNG, the treatment process includes three main steps: (1) biogas cleaning, (2) biogas upgrading, and (3) the compression of purified biogas to bioCNG. Figure 2b shows a simplified process flow for biogas cleaning, upgrading, and compression, with the parameter details summarized in Table S1 and the process details described in the SI.

### Biogas-to-PHB and Biogas-to-SCP Processes

An alternative to the use of biogas to produce bioCNG is to route the raw biogas for microbial conversion to valuable products, such as PHB and SCP. We developed the PHB production model (Figure 2c) and collected the input parameters from previously published studies.^21,38−41^ PHB is synthesized as intracellular storage granules under unbalanced growth conditions, that is, with excess carbon but deficient in key nutrients for cell replication (e.g., nitrogen and phosphorus).^38−40^ This microbial biosynthesis is an aerobic bioconversion process, including cell growth and PHB accumulation stages. The bacteria grow with sufficient carbon sources and nutrients during the cell growth stage, while a key nutrient (nitrogen herein) is limited during the PHB accumulation stage to stop the cell growth and accumulate PHB. Next, the PHB-rich cell mass is dewatered and mechanically disrupted to isolate the intracellular PHB granules from cells,^41^ followed by centrifugation to separate the PHB granules from cell debris. Finally, the separated PHB granules are dried to obtain pure marketable PHB power.

To produce SCP as the final product (rather than PHB), whole methanotrophic cells containing PHB are harvested from the aerobic bioconversion process (i.e., including both cell growth and PHB accumulation stages). In this case, the downstream processing is relatively simplified because the PHB isolation step is eliminated: the PHB-rich cell mass is dewatered, heat inactivated, and dried to produce the final SCP product (Figure 2d).^21,42^ The process details for the PHB and SCP production are included in the SI (Figure S2).

### Techno-Economic Analysis

Using the process configurations described above, each scenario was designed and simulated using the process simulation software *SuperPro Designer V11*. This software determines the required number and size of equipment based on operating conditions, performance requirements, and/or physical limitations on the available size. Each biorefinery was sized to process 2000 dry t of corn stover per day, with a delivered feedstock cost of $100/dry t.^30^ The annual operating time is 8410 h, and the plant life is 30 years. Incoming manure is conservatively assigned a cost based on its nutrient value, while food waste is assigned a tipping fee (revenue for the biorefinery rather than a cost). The MESP is reported in costs per gallon of gasoline equivalent ($/gge), adjusted using the higher heating value (HHV, 89 MJ/gallon). All costs were scaled and reported in 2020 U.S. Dollars. Unless otherwise specified, we determined the economics of ethanol production following the previously referenced NREL work,^13^ detailed in the SI (Tables S3 and S4). The biorefinery scale of 2000 dry t of corn stover per day was also based on the NREL study,^13^ which suggested that cost reductions due to economies of scale beyond this size would be modest, while feedstock transportation costs would increase as biomass is sourced from increasingly long distances. Furthermore, single-point sensitivity analysis was conducted to identify the most influential input parameters on the MESP (Figure S3).

### Life-Cycle Greenhouse Gas Emissions

The life-cycle GHG assessment scope is cradle-to-biorefinery gate with a functional unit of 1 MJ bioethanol produced, adjusted using HHV. The system boundary (Figure 1) includes all stages described above, including corn stover production and logistics, bioethanol production, and biogas conversion to bioCNG/PHB/SCP. System expansion is applied to include the emissions avoided due to displaced processes, including conventional manure management (i.e., direct manure land application where manure is collected and stored until land-applied), food waste landfilling, grid electricity offset, fossil CNG or diesel offset by bioCNG, fossil polymer offset by PHB, and fishmeal offset by SCP. All mass and energy balances for the simulated biorefineries were directly obtained from *SuperPro Designer*. Life-cycle inventory (LCI) data for chemicals, materials, and fuels were primarily assembled from widely used LCI databases including the Greenhouse gases, Regulated Emissions, and Energy use in Technologies (GREET) model,^43^ Ecoinvent,^44^ and Waste Reduction Model.^45^ We then used a hybrid LCA approach combining a process-based model with a physical unit-based input–output matrix (dataset, including the input–output table and impact vectors, presented in the SI) to calculate the life-cycle GHG emissions for each unit product/process.^36,37,46−48^ This choice of the model allows us to harmonize with commonly used models, such as GREET, where appropriate while also making the results more reproducible. The life-cycle GHG emission values and sources are provided in Table S5, and the details of the uncertainty analysis are in Table S6.

## Results and Discussion

### Adding Codigestion of Organic Wastes with BioCNG or PHB Production Can Improve Biorefinery Economics

**Figure 3:**
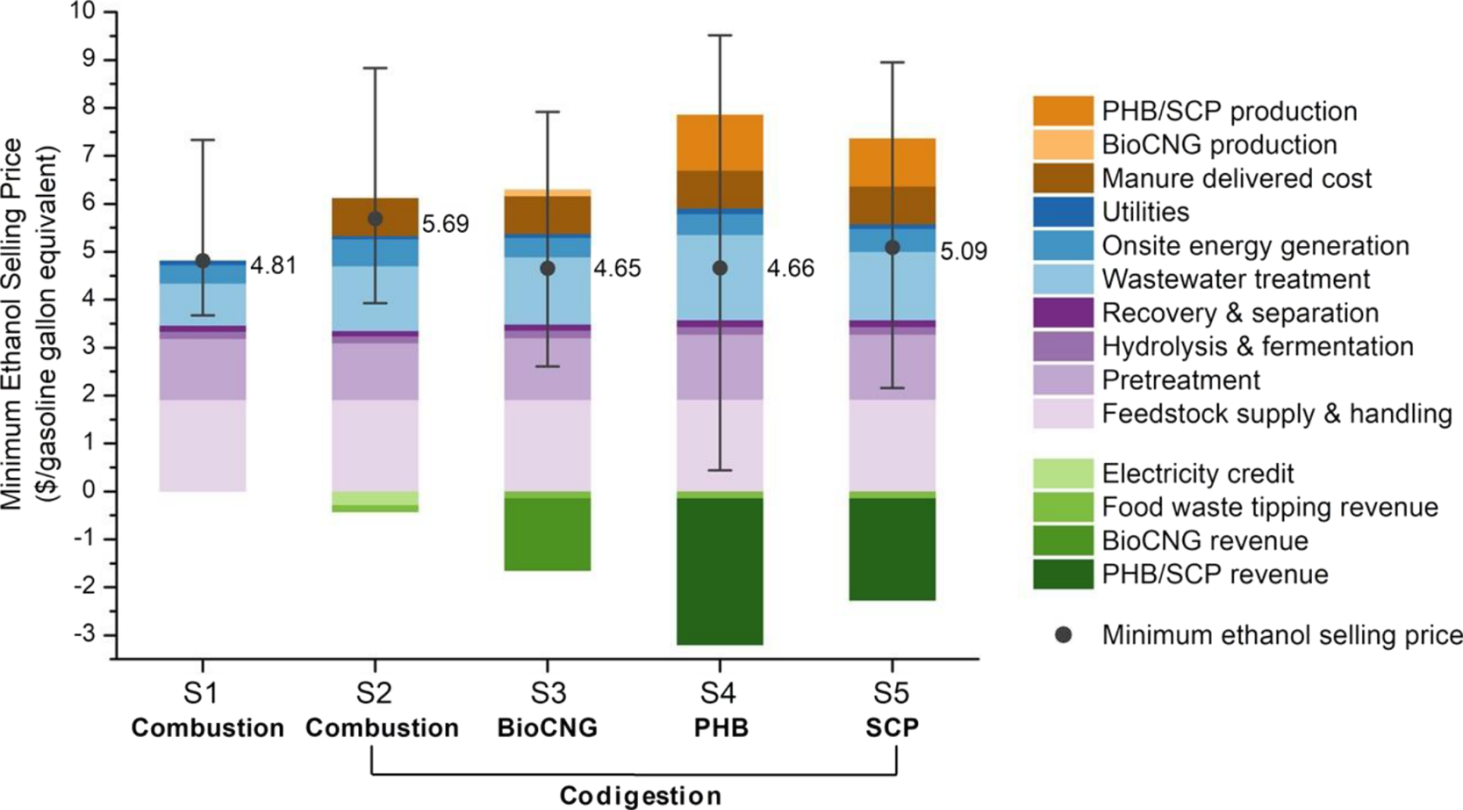
TEA results of biorefineries for bioethanol production incorporating codigestion of organic wastes and biogas utilization routes for bioCNG, PHB, and SCP production. The contribution to the MESP ($/gasoline gallon equivalent) is shown by process areas and credits (electricity export, food waste tipping revenue, and revenues from selling bioCNG, PHB, and SCP). S1: biorefinery with biogas onsite combustion. S2: integrated biorefinery with codigestion of organic wastes and biogas onsite combustion. S3: integrated biorefinery with codigestion of organic wastes and biogas upgrading to bioCNG. S4: integrated biorefinery with codigestion of organic wastes and biogas conversion to PHB. S5: integrated biorefinery with codigestion of organic wastes and biogas conversion to SCP. The amounts of organic wastes for codigestion are 4600, 1100, and 400 wet metric tons/day for hog manure, cattle manure, and food waste, respectively. The MESP values (labeled on the right of each bar) were determined using the baseline values of input parameters. Uncertainty bars represent the final MESP values for the pessimistic worst case and the optimistic best case considering the minimum and maximum values of key input parameters (Table S1). The numeric values for this figure are compiled in Table S7.

Favorable economics are a prerequisite to implementing any of the designs explored in this study, regardless of any environmental advantages. Codigestion of local organic wastes at a lignocellulosic biorefinery increases the scale of AD, and the incoming waste can serve as either a cost or a revenue, depending on what is accepted. In our analysis, the dominant local organic waste, manure, comes at a cost, while food waste can be a revenue source. The question is whether the tipping fee revenue and sales of final products (bioCNG, PHB, or SCP) outweigh the additional costs of increasing the AD capacity, sourcing manure, and downstream biogas upgrading or conversion. Different scenarios (S1–S5) are used to capture variations in the type and quantity of organic waste accepted for codigestion, as well as options for downstream biogas utilization, each resulting in a different MESP. We show in Figure 3 the contributions of each cost component (process areas and credits) to each scenario’s MESP, as well as pessimistic and optimistic cases to capture the uncertainty boundary. For comparison, the U.S. Department of Energy target for lignocellulosic ethanol is $3/gge ($2.05/gallon ethanol),^49^ which is close to recent wholesale gasoline prices.^50^

If biogas is combusted onsite, the results indicate that codigestion is likely not economically advantageous. The MESP in S1 (baseline) is $4.81/gge and increases to $5.69/gge in S2 (codigestion). This difference is driven by the additional WWT costs in the codigestion scenario ($0.88/gge in S1 to $1.35/gge in S2) and the fact that the waste intake will be dominated by hog and cattle manure, which we conservatively assume are a cost ($0.79/gge) rather than free or a source of revenue. The tipping fee revenue can be gained by accepting food waste, but typical food waste availability in candidate locations across the Corn Belt region is an order of magnitude smaller than manure availability. Another difference between S1 and S2 is that the S2 biorefinery scenario is capable of self-supplying all of its electricity needs, with 38% excess power exported to the grid due to the significant increase in the quantity of biogas (from 0.34 to 0.65 million Nm^3^/day). The MESP (S1) is sensitive to variations in the corn stover supply cost, solid loading rate during enzymatic hydrolysis, enzyme loading rate, sugar-to-ethanol conversion rate, and refining energy consumption during pretreatment (Figure S3a). Consistent with previous findings,^51^ the digester retention time is the most influential parameter (Figure S3b–e), and reducing the retention time from 25 to 15 days decreases the MESP in S2 from $5.69/gge to $5.22/gge. Strategies to reduce the retention time, such as utilizing substrates with high biodegradability, microbial immobilization systems, and high organic loading rate, might improve the economics of a digester.

The codigestion scenarios (S2–S5) all include the same organic waste intake. The results for S2 illustrate the limited economic benefits of generating additional biogas if that biogas will only be combusted onsite for heat and electricity. The next question is whether upgrading biogas to more valuable products is economically advantageous. The MESPs are $4.65, $4.66, and $5.09 per gge for bioCNG, PHB, and SCP production, respectively. All three scenarios (S3–S5) achieve lower MESPs than implementing codigestion with onsite combustion (S2). Both S3 and S4 achieve lower MESPs than the baseline S1 biorefinery, in which no organic waste is codigested ($4.81/gge). However, the relative advantage of converting biogas to higher-value products will likely grow in the long term because biorefineries that export power will face competition from renewables with a very low marginal cost of generation.^52^ The results are also sensitive to revenues generated from sales of bioCNG, PHB, and SCP. We assume that bioCNG, PHB, and SCP are sold at commodity prices of $0.81/kg,^53^ $4.75/kg,^38^ and $2/kg,^21^ respectively. These prices can fluctuate with many factors (oil price volatility, uncertain demand, targeted application sectors, policy incentives, etc.). For example, the PHB selling price is the most sensitive factor to the MESP in S4 (Figure S3d); a greater price of $7/kg substantially decreases the MESP from $4.66/gge to $3.21/gge, whereas a lower price of $2.5/kg elevates the MESP to $6.11/gge. Identifying marketable end-uses with higher selling prices of PHB (e.g., for some more advanced applications) is vital for attaining improved process profitability.

Due to economies of scale, a facility that converts a smaller volume of biogas than what was modeled in this study will face increased unit production costs (and vice versa). The unit production cost of a biogas-converted product was calculated using amortized capital expenditures and net operating costs in its respective process area (i.e., comprising the process units that convert biogas to bioCNG or PHB/SCP). As a comparison, the bioCNG production cost in this study ($0.07/kg) is lower than the estimate ($0.21/kg) in our previous work (Yang et al.)^36^ that employed the same biogas upgrading method (i.e., membrane separation) but had a smaller production scale (∼17 million kg CH_4_/year relative to 78 million kg CH_4_/year herein). Sheets and Shah reported a bioCNG production cost of $0.36/kg at a scale of ∼22 million kg/year,^54^ which is higher than Yang et al.’s result ($0.21/kg) because more costly pressure swing adsorption was used for biogas upgrading.^55,56^ In addition, the production cost estimated here for PHB ($1.75/kg at a scale of 26.9 million kg/year) is significantly lower than those (> $15/kg) on much smaller scales (e.g., 0.5 million kg/year)^57^ but also lower than that ($4.9/kg) in a previous study by Levett et al.,^38^ who evaluated larger-scale production of PHB (100 million kg/year). The higher PHB production cost in Levett et al.’s study than ours could be primarily attributed to their use of purchased methane and utilities as well as solvent extraction downstream processes that are less economically effective due to solvent demand and recovery.^39,58^

All these biogas utilization scenarios (S3–S5) require imported electricity to meet process energy needs. In S3 (which produces bioCNG), 57% of the onsite electricity demand can be met with renewable onsite energy generation (combustion of lignin and other residues), while only 34% of the onsite electricity demand can be satisfied in S4 (PHB) and S5 (SCP). This difference is driven by the more energy-intensive processes required to produce PHB and SCP, where electrical energy accounts for about half of the total electricity consumption in the biorefinery and is mainly attributed to bioreactors, mechanical disruption, and gas compressors. The differences in energy balances across each biorefinery scenario have a limited impact on the MESP. However, these differences can become more important in determining the life-cycle GHG footprints.

### Impact of Organic Waste Tipping Fees

**Figure 4:**
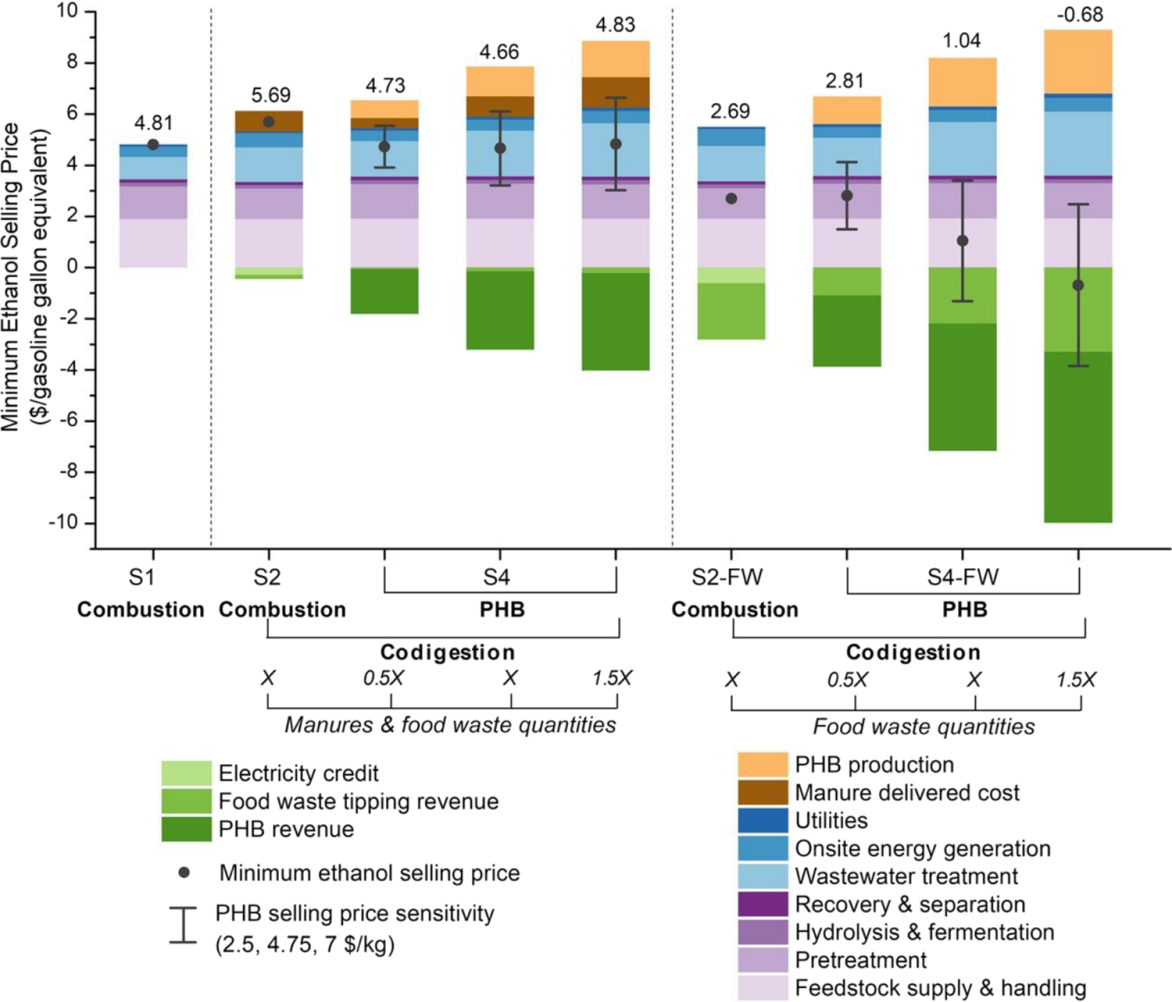
TEA results of the biorefineries incorporating codigestion of organic wastes with varying types and quantities and the biogas utilization route to PHB. The contribution to the MESP ($/gasoline gallon equivalent) is shown by process areas and credits (electricity export, food waste tipping revenue, and PHB selling revenue). S1: biorefinery with biogas onsite combustion. S2 and S2-FW (food waste): integrated biorefinery with codigestion of organic wastes and biogas onsite combustion. S4 and S4-FW: integrated biorefinery with codigestion of organic wastes and biogas conversion to PHB. Blends of hog manure, cattle manure, and food waste are considered in S2 and S4, while only food waste is considered in S2-FW and S4-FW. The quantities of organic wastes vary in S4 and S4-FW; a decrease by half and an increase by 50% relative to average resource availability (X, a total of 6100 wet metric tons/day) were modeled for comparison. The MESP values (labeled on top of each bar) were determined using the baseline values for input parameters. Uncertainty bars represent the sensitivity of the PHB selling price. The numeric values for this figure are compiled in Table S8.

As discussed previously, different types of organic wastes may either be purchased, delivered at no cost, or even accepted along with a tipping fee comparable to what a landfill would require to accept the waste. Taking the biogas-to-PHB scenario as an example, we further explore how different combinations of the organic waste intake impact the MESP (Figure 4). The quantity of waste loaded into the digester impacts AD capital costs and the quantity of biogas generated. The types of organic wastes codigested will impact biogas yields as well, although this is of secondary importance compared to the variation in costs/revenues for accepting them. Using an S4 biorefinery configuration (codigestion producing PHB from biogas) as a test case, we first varied the total quantity of organic waste accepted while keeping the ratio of wastes constant (for mixed manures and food waste). Specifically, the total quantity decreases by half (to 3050 t/day) or increases by 50% (to 9150 t/day) on the basis of average resource availability (6100 t/day). For perspective, 9150 t/day of wet organic wastes is roughly equivalent on a dry mass basis to the daily intake of corn stover in our model. In a separate set of scenarios, we explored the potential to accept the same total quantities of waste but source only food waste (accompanied by a tipping fee) for codigestion.

If the breakdown of the organic waste intake is held constant (primarily manure, with a much smaller quantity of food waste), the results in Figure 4 suggest that the costs of handling additional waste and the revenues to be generated from selling PHB are fairly well balanced. Cutting the waste intake in half actually increases the MESP by 1.5%, while increasing the intake by 50% increases the MESP by 3.6%. However, for food waste-only scenarios (the S4-FW set scenarios in Figure 4), the tipping fee revenue reduces the MESP for all scenarios and creates a strong economic incentive to accept larger quantities of waste. A significant decrease in the MESPs is observed with increasing scale of AD facility by 50%, achieving a negative MESP ($–0.68/gge). PHB revenues also increase when the biorefinery takes in exclusively food waste rather than the more manure-dominated mix because food waste is estimated to result in higher biogas yields (Table S2). However, the degree to which the food waste-only scenarios are realistic, and whether such waste will indeed come with a tipping fee, will vary by region. Cleaner food waste may have alternate markets as animal feed, for example. More contaminated food waste may require additional pretreatment that is not modeled in this study, thus increasing facility costs. Nevertheless, the findings here highlight the essential role of tipping fees in driving the economics, particularly if bioethanol must sell for under $3/gge to gain a significant market share.

### Codigestion Scenarios Can Achieve Net Negative GHG Emissions

**Figure 5:**
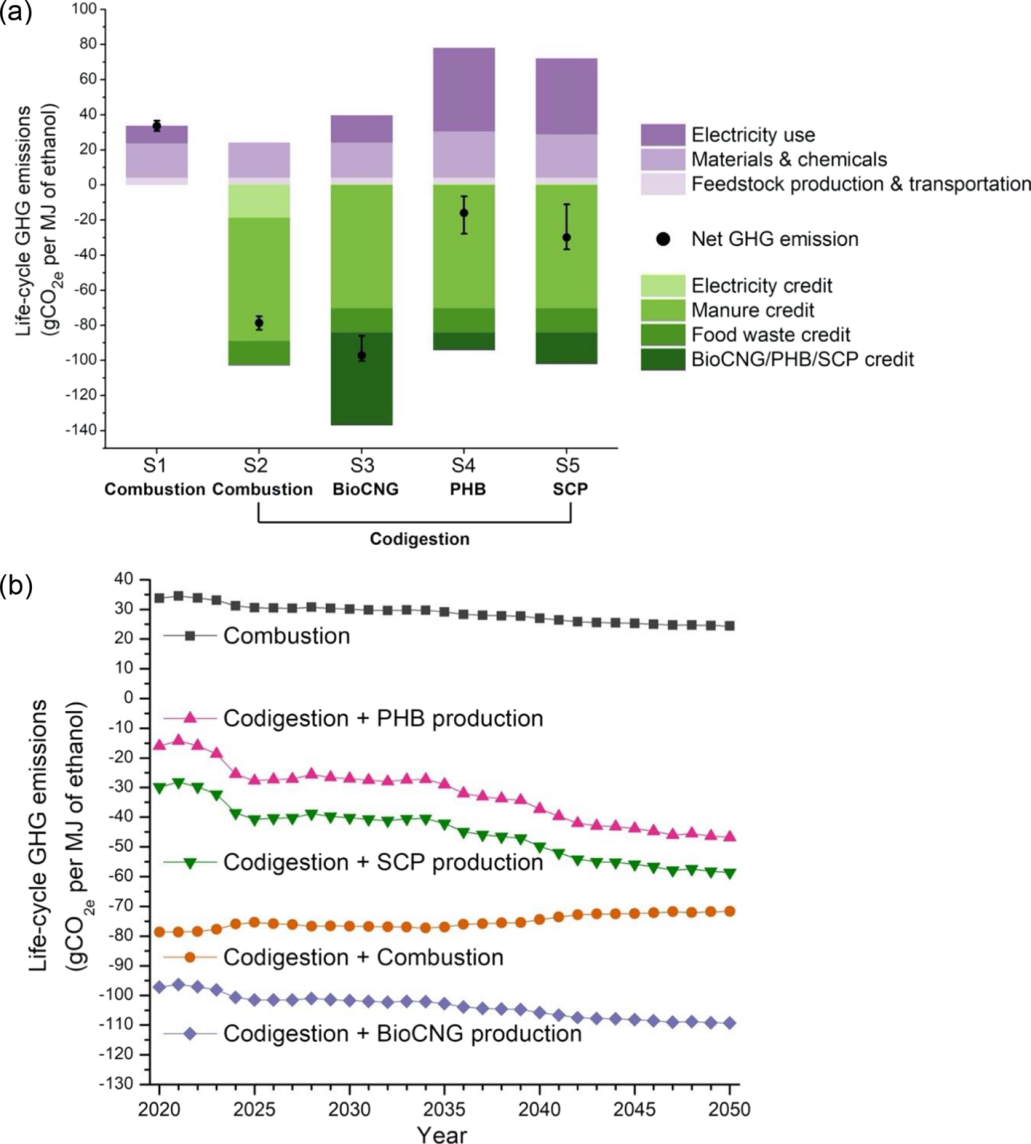
Life-cycle GHG emissions for different scenarios. (a) Contribution to the GHG emissions is shown by input categories and offset credits (outlined in Figure 1). S1: biorefinery with biogas onsite combustion. S2: integrated biorefinery with codigestion of organic wastes and biogas onsite combustion. S3: integrated biorefinery with codigestion of organic wastes and biogas upgrading to bioCNG. S4: integrated biorefinery with codigestion of organic wastes and biogas conversion to PHB. S5: integrated biorefinery with codigestion of organic wastes and biogas conversion to SCP. The amounts of organic wastes for codigestion are 4600, 1100, and 400 wet metric tons/day for hog manure, cattle manure, and food waste, respectively. The GHG emissions were determined using the baseline values for inputs and offset credits (with bioCNG for offsetting diesel fuel). Uncertainty bars capture variations in all inputs and offset credits. The numeric values for (a) are compiled in Table S9. (b) Change in the life-cycle GHG emissions as a function of the electric power projections (2020–2050). Projection data (Figure S5) for two electricity subregions were considered to represent the direct electricity source for the Corn Belt region, including midcontinent independent system operator west and central. The average U.S. electricity mix was considered as the source of indirect (upstream) electricity.

Diverting organic waste for codigestion in biorefineries produces substantial GHG benefits, regardless of the specific scenario, as shown in Figure 5a. Emission avoidance credits are assigned to manure and food waste commensurate with the business-as-usual treatment (land application for manure and landfilling for food waste). The net GHG footprint in the baseline scenario (S1) is 34 gCO_2e_/MJ, reaching a reduction of 63% relative to gasoline (93 gCO_2e_/MJ^59^). The gasoline GHG footprint includes CO_2_ emissions from gasoline combustion, whereas ethanol combustion emissions are excluded because they are biogenic; in both cases, transportation from the production facility to fueling stations has been excluded. This 63% GHG reduction satisfies the emission reduction target (60%) set by the Renewable Fuel Standard program for cellulosic fuels to qualify for Renewable Identification Number (RIN) credits.^3^ The DMR pretreatment process, which was selected based on the expectation that it would produce fewer inhibitors that could jeopardize AD operations, does result in greater chemical and electricity-related emissions. A lower GHG footprint for the baseline scenario (∼28 gCO_2e_/MJ) could be achieved by employing other pretreatment methods such as IL pretreatment.^36^

Codigestion of organic wastes results in an avoidance of 70 gCO_2e_/MJ (manure) and 14 gCO_2e_/MJ (food waste) by diverting organic wastes from more GHG-intensive disposal options. If biogas is combusted onsite (S2), the export of excess electricity leads to a GHG offset credit of 19 gCO_2e_/MJ, although these results will vary depending on the biorefinery’s local grid mix. The waste-related GHG credits combined with electricity export credits result in a negative footprint totaling −79 gCO_2e_/MJ for this codigestion–combustion scenario (S2). Biogas upgrading to bioCNG (S3) requires electricity imports from the grid but earns a larger emission offset credit (−53 gCO_2e_/MJ) if bioCNG can be used to offset diesel use in heavy-duty vehicles. Ultimately, S3 results in the lowest GHG footprint (−97 gCO_2e_/MJ). Offsets will be smaller if upgraded biogas is used to displace fossil natural gas, which is less GHG-intensive than diesel fuel.^46^ Sahoo and Mani investigated the environmental impacts of different AD technologies producing bioCNG from dairy manure, food waste, and miscanthus biomass feedstocks; negative GHG emissions were achieved for all scenarios mostly due to credits from displaced fossil fuel and diverted organic wastes.^60^ Converting biogas to PHB and SCP both result in negative, but higher, GHG footprints: −16 gCO_2e_/MJ for the biogas-to-PHB scenario (S4) and −30 gCO_2e_/MJ for the biogas-to-SCP scenario (S5). The emission footprints are not as strongly negative because of smaller offset credits assigned to PHB and SCP, combined with the fact that producing SCP and PHB requires more electricity than what is consumed in the bioCNG scenario. The PHB offset credit is based on a range of plastics commonly used in packaging, which are produced from oil and gas,^24^ resulting in life-cycle GHG emissions between 1.60 and 2.92 kgCO_2e_ per kg of fossil-based polymer.^43^ The resulting PHB offset credit is 10 gCO_2e_/MJ ethanol. Because the SCP yield is ∼65% higher than that of PHB, more emissions (18 gCO_2e_/MJ) are avoided for offsetting the production of fishmeal (1.97 kgCO_2e_/kg fishmeal^61^). Similar findings are obtained in the life-cycle fossil energy demand for the five scenarios, detailing the depletion of petroleum, natural gas, and coal (Figure S4).

### Impact of Grid Decarbonization on Life-Cycle GHG Emissions

The life-cycle GHG results presented in this study are sensitive to assumptions about the electricity grid mix. Future projections through 2050 suggest that the share of renewable electricity generation is likely to grow.^52^ The more electricity-driven scenarios, biogas-to-PHB (S4) and biogas-to-SCP (S5), will be disproportionally impacted by this change. In 2020, the fuel types for electricity generation in the Corn Belt region included coal (51%), wind (19%), natural gas (15%), and nuclear (13%). By 2050, the U.S. Energy Information Administration high renewable energy penetration scenario includes dramatic increases in wind (53%) and solar (14%) power at the expense of coal (18%) and nuclear (1%) (Figure S5). The impact of projected grid decarbonization on our GHG results is shown in Figure 5b, and the impacts on the fossil energy demand are shown in Figure S6. The net GHG footprint for the PHB and SCP scenarios is projected to decrease by ∼30 gCO_2e_/MJ by 2050, and the bioCNG production scenario will decrease by ∼13 gCO_2e_/MJ. The transition to a lower-carbon grid is occurring more rapidly in some regions than others. The Midwest grid mix is relatively carbon-intensive,^43^ and other lower-emitting U.S. grid regions can offer advantages for biorefineries seeking to scale up the more electricity-intensive PHB or SCP processes.

### Carbon Mitigation Costs for Integrated Biorefineries

**Figure 6:**
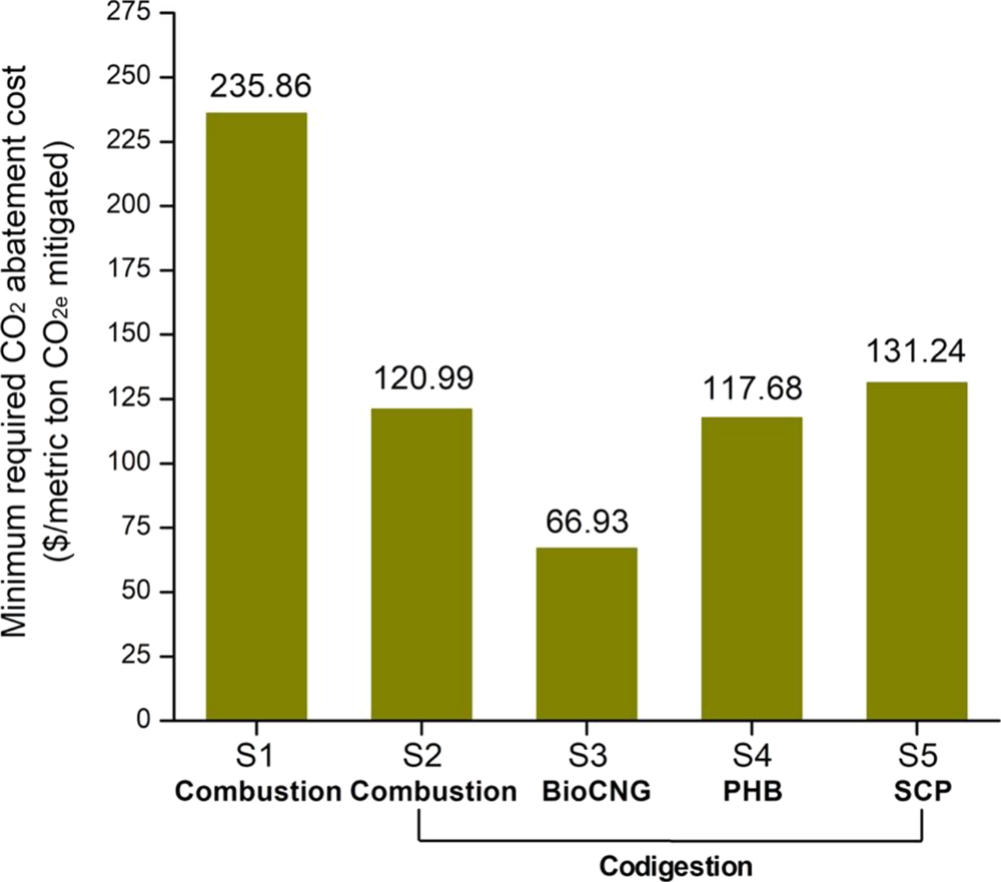
Minimum required price per metric ton of CO_2e_ mitigation at a fixed bioethanol selling price ($3/gasoline gallon equivalent, $2.05/gallon ethanol) for different scenarios. S1: biorefinery with biogas onsite combustion. S2: integrated biorefinery with codigestion of organic wastes and biogas onsite combustion. S3: integrated biorefinery with codigestion of organic wastes and biogas upgrading to bioCNG. S4: integrated biorefinery with codigestion of organic wastes and biogas conversion to PHB. S5: integrated biorefinery with codigestion of organic wastes and biogas conversion to SCP.

Combining life-cycle GHG emissions and costs into a single metric can be a useful way to evaluate different options with economic and environmental tradeoffs. The cost of GHG mitigation can also be compared to the value of mitigation on regulated carbon markets. In addition to RINs, California’s Low Carbon Fuel Standard (LCFS)^62^ and Oregon’s Clean Fuels Program (CFP)^63^ are two leading clean fuels policies that are currently implemented in the U.S., while similar programs are being developed in other states (e.g., Midwestern states and New York).^64^ Credits are generated according to the reductions in the carbon intensity of biofuels relative to the baseline conventional fuels being displaced. Combining the results of MESPs (Figure 3) with life-cycle GHG emissions (Figure 5a), we calculated the cost per metric ton of avoided CO_2e_ that is needed to reach the desired MESP target ($3/gge to represent cost parity with petroleum).^36,47,49^ The GHG mitigation costs for S1–S5 are $236, $121, $67, $118, and $131/t CO_2e_, respectively (Figure 6). Unsurprisingly, the biogas-to-bioCNG scenario (S3) offers the lowest carbon mitigation cost. All four codigestion scenarios offer a lower cost of carbon mitigation when compared to the base case (S1). The results for PHB (S4) and SCP (S5) are sensitive to their respective assumed selling prices, so a price premium for either product will drive down their carbon mitigation costs. For comparison, the LCFS credits ranged from ∼$140 to ∼$220/t CO_2e_ in 2020 with an annual average credit of ∼$200/t CO_2e_.^62^ The CFP credits averaged in 2020 was ∼$128/t CO_2e_ for a range of ∼$111 to ∼$159/t CO_2e_.^63^ In addition, the U.S. Environmental Protection Agency and other federal agencies use the estimated social cost of carbon to value the climate impacts of rulemaking; the high impact value for 2020 was $123/t CO_2e_.^65^ Our costs of carbon mitigation for the integrated biorefinery scenarios (S2–S5) are comparable to these market values, which is an encouraging result.

## Implications and Future Work

The role of bioeconomy in climate change mitigation is a subject of intense debate, with many competing visions for how (or whether) to scale up the production of biobased fuels and chemicals. We propose re-envisioning biorefineries as critical infrastructure hubs, where agricultural residues and mixed wastes can be converted to fuels, plastics, and even feed products. Although there are still technical challenges associated with cost-effectively converting lignocellulosic biomass to fuels, the results are encouraging. Biorefineries that codigest high-moisture organic waste alongside process wastewater and upgrade biogas to bioCNG, PHB, or SCP achieve substantial reductions in the cost per metric ton of CO_2e_ mitigated. The bioCNG scenario proved most attractive on an economic and life-cycle GHG basis, although widespread vehicle electrification may reduce the market demand for bioCNG as a transportation fuel. PHB and SCP production, conversely, will only become more attractive with long-term shifts toward electrification and a decarbonized grid.

The impacts of implementing the biorefinery designs presented here extend well beyond costs and GHG emissions. PHB plastic products are biodegradable; by replacing conventional plastic packaging with these materials, it is possible to reduce the generation of persistent plastic waste. Incidentally, PHB can rapidly degrade under anaerobic conditions; although composting has been regarded as a common end-of-life treatment method for biodegradable plastics, PHB waste could itself be recycled back to be treated using AD.^24^ Not all non-GHG impacts will be positive, however. Increasing manure and food waste intake may cause local permitting challenges based on increased nutrients in discharged wastewater, even if diverting manure from storage lagoons reduces eutrophication impacts elsewhere.

It will also be important to be mindful of the residual solids remaining after AD. We assumed that the solid digestate can be combusted to provide heat and electricity onsite; however, the solid digestate may be suitable for direct land application on local farms.^66,67^ Land-applying digestate returns nutrients to the cropland and can displace some use of inorganic fertilizers like urea. Compared to untreated manure, digested manure has lower GHG emissions from storage, lower N_2_O emission during land application, and higher mineral-N content to maintain an equal or higher crop yield without negative effects on the soil.^66,68^ Also in some states (e.g., Pennsylvania, Maryland, and Virginia), nutrient credits are applicable in the agriculture sector where nutrient loading is regulated through trading markets to protect water quality and ecosystem, thus creating additional revenues for the AD treatment of manure relative to the direct use of untreated manure for land application.^67^ These economic and environmental advantages of land-applying solid digestate may incentivize biorefinery owners to generate less energy onsite. The result would be a need for more electricity imports but the addition of a potential offset credit for avoided fertilizer use. In addition to solid digestate, codigestion of high-moisture organic wastes at biorefineries may generate excess treated water in the WWT area. This recycled wastewater may replace freshwater in agricultural and landscape irrigation, which will not meaningfully impact our economic or GHG results but can alleviate pressure on freshwater resources in water-scarce regions.

There are potentially attractive options that were outside the scope of this study but would be worthwhile to explore in future work. For example, biogas can be converted through gas fermentation to ethanol, commodity solvents, or more advanced fuels.^69^ Another interesting subject for future work is about the capture and sequestration of biogenic CO_2_-containing streams. Biorefineries generate CO_2_-rich streams, including the gaseous streams from fermenters, aerobic WWT basins, waste CO_2_ after biogas upgrading/conversion, and flue gas from the CHP units. In particular, the streams from biogas upgrading and ethanol fermentation both produce relatively pure CO_2_ streams that can be directly transported and sequestered without additional treatment if geologic formations are available within a reasonable distance. Large-scale bioenergy with carbon capture and sequestration is considered essential to most climate stabilization scenarios to compensate for difficult-to-decarbonize sectors and is likely to be the subject of continued research and policy interest.^70^ Although there will be no one-size-fits-all solution, this study offers a broader solution space for lignocellulosic biorefineries that will hopefully offer economic, environmental, and community benefits beyond what can be achieved with today’s biorefineries.

## Supporting Information Available

Input data and method details for process modeling, TEA, LCA, and sensitivity/uncertainty analyses; numeric values for Figures 3, 4, and 5a; electric power projections; and life-cycle fossil energy demand results (PDF)
Input–output table for GHG emissions (XLSX)
