# Supplementary material for: Co-Processing Agricultural Residues and Wet Organic Waste Can Produce Lower-Cost Carbon-Negative Fuels and Bioplastics
Source: Environ Sci Technol. 2023 Feb 7;57(7):2958–69. doi: 10.1021/acs.est.2c06674 (PMC9948286; doi:10.1021/acs.est.2c06674)
Supplement: Supplementary file 1 — es2c06674_si_001.pdf [file es2c06674_si_001.pdf]

SUPPORTING INFORMATION

Co-processing agricultural residues and wet organic waste can produce lower-cost carbon-negative fuels and bioplastics

Yan Wang, [1,2,3] *Nawa R. Baral,*[2,3] *Minliang Yang,*[2,3] *Corinne D. Scown**[1,2,3,4]

[1]Energy & Biosciences Institute, University of California, Berkeley, Berkeley, California 94720, United States

[2]Life-cycle, Economics, and Agronomy Division, Joint BioEnergy Institute, Emeryville, California 94608, United States

[3]Biological Systems & Engineering Division, Lawrence Berkeley National Laboratory, Berkeley, California 94720, United States

[4]Energy Analysis & Environmental Impacts Division, Lawrence Berkeley National Laboratory, Berkeley, California 94720, United States

Pages S1- S33

Tables S1-S14

Figures S1-S6

ADDITIONAL METHOD DETAILS

Corn stover production and logistics

The composition of corn stover used for our analysis is shown in Table S1. The supply logistics cost of corn stover (20% moisture) is estimated to be \$100.2/dry t (see Baral et al.' study[1] for method details), including the costs of establishment (machinery, labor, fuel, and land rent), nutrient replacement and herbicide/pesticide application, machinery operations (windrowing, baling, stacking), and transportation by truck from the field to the biorefinery in the form of bales.

Conversion of biomass to bioethanol

Figure 2 shows the biorefinery process. Figure 2a provides the full details of Scenario 1, while Figures 2b-d show just the variations in the downstream anaerobic digestion and biogas upgrading sections.

The DMR pretreatment process includes the following steps:[2] corn stover is deacetylated with an alkaline loading of 50 kg NaOH per bone-dry metric ton (bdt) at 80 °C for 2 h; the slurry is dewatered to 40% total solids using screw press, the cake is screw conveyed to the mechanical refiner (grinding) with an energy consumption of 200 kWh/bdt while the filtrate goes to the WWT section. The pretreated biomass is converted to sugars through enzymatic hydrolysis and further fermented to ethanol. Distillation and molecular sieve adsorption are used to recover 99% of the ethanol from the fermentation broth with a purity of 99.5%. The stillage after ethanol recovery is transferred to the WWT section. Wastewater is treated using AD, aerobic activated-sludge lagoon, and reverse osmosis. The effluent can be recycled for reuse as process water. The biogas produced from AD, the dewatered WWT sludge (including the solid digestate from AD), as well as the remaining polysaccharides and lignin separated from ethanol recovery, are delivered to the onsite CHP unit for steam and electricity generation. Operating conditions for all process areas are compiled in Table S1.

Organic waste availability for codigestion

The compositions of hog manure, cattle manure, and food waste are summarized in Table S2, including the compiled results of previous studies for total solid (TS), volatile solid (VS), lipid, protein, and carbohydrate. Based on the average TS for hog manure (11%), cattle manure (14%), and food waste (20%), their average amounts within an 80 km of farm-to-biorefinery distance in the Corn Belt region are approximately 4,600, 1,100, and 400 t per day on a wet basis, respectively. A similar logistics model used for corn stover[1] was used to estimate the truck transportation cost of organic wastes (Table S1).

Prediction of biogas production from anaerobic codigestion

The organic components (VS) to represent manures and food waste are lipid ($C_{57}H_{104}O_6$), protein ($C_5H_7NO_2$), and carbohydrates ($C_6H_{10}O_5$), with the residue denoted as lignin ($C_8H_8O_3$ for vanillin).[3] The theoretical reaction equation of biogas production (equation 1) is shown as follows:[4]

$$C_nH_aO_bN_xS_y + \left(n - \frac{a}{4} - \frac{b}{2} + \frac{3x}{4} + \frac{y}{2}\right) H_2O \rightarrow \left(\frac{n}{2} - \frac{a}{8} + \frac{b}{4} + \frac{3x}{8} + \frac{y}{4}\right) CO_2 + \left(\frac{n}{2} + \frac{a}{8} - \frac{b}{4} - \frac{3x}{8} - \frac{y}{4}\right) CH_4 + xNH_3 + yH_2S \quad (1)$$

For the two streams resulting from ethanol production (i.e., the pressed filtrate after DMR pretreatment and the stillage after ethanol recovery), we used a previously reported maximum methane yield of 356 mL CH_4/g VS for alkaline (NaOH) pretreated lignocellulosic feedstocks at a severity factor of 1.5 (Figure S1). The average methane yields for hog manure, cattle manure, and food waste are 278, 164, 343 mL CH_4/g VS, respectively (Table S2). For all scenarios, sufficient biogas is diverted for combustion to satisfy the onsite heat demand, while the electricity demand, if not fully satisfied by onsite generation, can be met with imports from the grid.

Biogas-to-BioCNG process

First, biogas cleaning (i.e., the removal of trace impurities) is mandatory to prevent the corrosive effects on storage tanks, pipelines and engines.[5] H_2O and H_2S are two typical impurities to be removed during the cleaning step.[6,7] In general, two steps of water vapor removal or desulfurization, namely primary and precise methods, are required to meet the gas quality for transport fuel or pipeline.[7] We selected the most applied techniques to separate H_2O and H_2S from biogas. Specifically, condensation is used as the pretreatment method to remove large amount (75%) of H_2O followed by the adsorption method on silica gel[8] to reduce the H_2O level to < 0.0012 mol%. H_2S is partially removed in the digester through the addition of $FeCl_3$, which removes sulfide by precipitating it out as FeS or oxidizing it to elemental sulfur, to reach an H_2S level less than 0.01 mol% and remaining H_2S is removed by high efficiency adsorption (< 0.0003 mol% H_2S) on activated carbon.[6] A separate cleaning step for NH_3 (another problematic contaminant) is not necessary because it can be eliminated through biogas drying or upgrading.[5] After biogas cleaning is complete, the biogas upgrading step removes CO_2 to reach the desired biogas purity (> 95% CH_4), thus increasing the calorific value. Based on our previous work,[9] we employed multi-stage membrane separation process for biogas upgrading to reach a high CH_4 recovery efficiency. Membrane separation is a mature technique to separate CH_4 from CO_2 that generally has lower investment and operational costs and does not require chemical or antifouling agents compared to other upgrading techniques (e.g., water/chemical scrubbing, pressure swing adsorption, cryogenic separation).[5] The upgraded biogas is estimated to have a CH_4 purity above 99% assuming a 5% CH_4 loss to the permeate. In addition, the separated CO_2-rich stream cannot be directly released to the atmosphere due to the presence of CH_4, particularly for the membrane technologies with > 1% CH_4 loss.[7] Off-gas treatment is commonly needed such as regenerative thermal oxidation, catalytic oxidation, co-firing in combustion engines, or using biofilters.[7] We haven't explicitly designed this step and simply assume the off-gas is sent to the onsite CHP unit for co-firing. Yet, the practical operation needs to ensure the minimum turbine calorific value is reached while using mixed energy carrier inputs. Finally, the purified biogas is compressed to 220 atm and stored.

Biogas-to-PHB and biogas-to-SCP processes

This microbial biosynthesis of PHB is an aerobic fermentation process, the enriched oxygen is supplied to bioreactors by using pressure swing adsorption for a feed air stream. The mixed *methanotroph* culture is used under non-sterile working condition due to its stable populations and ability to self-regulate.[10] The nutrients contain nitrogen (NH_4OH), phosphorus (KH_2PO_4), and trace metal salts (simply represented by $MgSO_4$ in the model given its dominance, ~80% of the total mass[10]). The bioreactors are operated at a temperature of 38 °C.[10,11] There are two stages for the fermentation process, cell growth and PHB accumulation. During cell growth stage, the bacteria grow with sufficient carbon source and nutrients for 48 h to obtain

a cell density of 20 g/L, while a small quantity of PHB (3 wt.% cell mass) is stored.[10,11] During PHB accumulation stage, nitrogen is limited so that the cells stop growing and start to accumulate PHB inside the body for 48 h to reach a cell density of 50 g/L containing 60 wt.% PHB.[10,11] The mass balances for the bioreactors are based on the following equations for cell growth (equation 2) and PHB production (equation 3),[10] where the CH_4 utilization efficiency is assumed to be 90%.[12]

$$2.46\ CH_4 + 0.25\ NH_4OH + 3.85\ O_2 \rightarrow Cell\ mass(CH_2O_{0.5}N_{0.25}) + 4.53\ H_2O + 1.46\ CO_2 \quad (2)$$

$$2.5\ CH_4 + 3.875\ O_2 \rightarrow PHB(CH_{1.5}O_{0.5}) + 4.25\ H_2O + 1.5\ CO_2 \quad (3)$$

The needs for phosphorus and trace metal salts are assumed to be 3 wt. % (on the basis of P) and 4 wt.% (on the basis of $MgSO_4$) of the cell mass, respectively.[10] Similar to the off-gas treatment for biogas upgrading, the flue gas from the bioreactors that still contains a small amount of CH_4 is sent to the onsite CHP unit. Next, the PHB-rich cell mass is dewatered and mechanically disrupted to isolate the intracellular PHB granules from cells,[13] followed by centrifugation to separate the PHB granules from cell debris. The non-PHB cell mass is delivered to the AD facility. Finally, the separated PHB granules are dried to obtain pure marketable PHB power. Downstream processing ultimately achieves a PHB recovery rate of 90%.[14] The water recovered from dewatering can be recycled for the growth media preparation.

Intracellular PHB increases the value of the SCP because of its observed prebiotic effects when used as a feed supplement, an alternative to antibiotics.[15] We modeled the production of PHB-containing SCP for scenario comparison in the main text. Alternatively, the cells harvested after the first fermentation stage (i.e., cell growth) can be also used as SCP, although they will result in a product that contains less PHB and will thus be less valuable. In this scenario, the PHB accumulation stage is excluded and the cell density in the cell growth bioreactor is increased to 50 g/L. We have included the cost results for this simplified process configuration (where cells are harvested after the first fermentation stage) in Figure S2. Assuming the same selling price of SCP, the production of PHB-deficient cells leads to a lower MESP ($4.75/gge) compared to the production of PHB-rich cells ($5.09/gge), owing to the reduction in the production cost and the increase in the cell mass yield. Yet, in view of the antibiotic value of intracellular PHB,[15] we might expect a higher selling price for PHB-rich SCP, which in turn will increase its production profitability.

Techno-economic analysis

Extensive details on process parameters and assumed costs are available in Table S1. Incoming manure is conservatively assigned a cost based on its nutrient value, with an average value of $60/dry t and a low and high of $40 and $80 per dry t, respectively. The cost of sourcing manure is likely to vary considerably depending on whether it is hauled from a confined animal feeding operation (CAFO) or collected from grazed animals, as well as local demand for manure as fertilizer. Food waste is assigned a tipping fee (revenue for the biorefinery, rather than a cost) based on average landfill tipping fees in the U.S. Midwest, with an average of approximately $52/wet t and a low and high of $42 and $68 per wet t. Transportation costs were separately estimated for hog and cattle manure, as well as food waste, and are detailed in Table S1.

After executing the mass and energy balances and determining the capital and operating costs, the discounted cash flow analysis was conducted to calculate the MESP, which corresponds to the selling price needed for ethanol to reach a zero net present value (NPV) with an internal rate of return of 10% and an income tax rate of 35%. It is important to note that the corporate tax rate is subject to change, and was reduced to 21% in the U.S. as of 2017. To ensure

that our results are comparable with other biofuel TEAs, many of which have used a 35% tax rate, we have opted to use the older value.

The capital cost for equipment was determined based on the equipment purchase cost and the installation cost (a multiple of the purchase cost using an average installation factor of 1.7). The total capital cost was further determined by adding other direct costs (warehouse, site development, additional piping), indirect costs (e.g., home office and construction, project contingency), and fixed costs (land, working capital). The details to calculate each capital item are provided in Table S3. The total operating costs include fixed costs (labor, overhead items) and variable costs (raw materials, waste disposal, imported electricity, coproduct credits). The variable operating costs were directly obtained from the software upon balancing mass and energy in each model. The labor cost was estimated by multiplying the total number of employees in each biorefinery scenario with associated salaries. The estimates for fixed operating costs are shown in Table S4. The unit production costs for bioCNG, PHB, and SCP were calculated using amortized capital expenditures and net operating costs in the process areas of bioCNG, PHB, and SCP production. All costs were scaled and reported in 2020 U.S. Dollars. This choice allows us to avoid incorporating potentially temporary price increase that have resulted from pandemic-related supply chain disruptions and labor shortages, although if those more recent price increases persist, future TEAs will need to ensure that input parameters are adjusted appropriately.

For the processes in our models that were not incorporated in the original NREL corn stover-to-ethanol process (i.e., bioCNG, PHB, or SCP production), two differences for estimating their capital and operating costs are: i) the equipment purchase costs for these new areas were estimated using the built-in model in *SuperPro Designer*; and ii) eleven employees were added to operate each of these areas (Table S4).

Table S1 summarizes the baseline, minimum, and maximum values of input parameters, while the minimum and maximum values were used to explore their impact of uncertainty on MESP.

Life-cycle greenhouse gas emissions

In this analysis, corn stover is assigned the marginal increase in fertilizer application and on-farm fuel use resulting from its collection. All other on-farm impacts are attributed to the main product (corn grain). Life-cycle GHG impacts were assessed in terms of CO_{2e} using the latest Intergovernmental Panel on Climate Change 100-year global warming potential factors for fossil CO_2 (1), CH_4 (30), and N_2O (265). Emissions related to the biorefinery infrastructure (construction, maintenance, decommissioning) were excluded as these are negligible over long lifetime of industrial plants.

Based on the assumption that the biorefinery is located in the Corn Belt region, we assume the direct electricity is imported from or exported to two electricity subregions, Midcontinent Independent System Operator West (MISW) and Central (MISC). The overall life-cycle GHG emissions footprint from electricity generation was determined by combining the percent distribution of fuel types used in these two regions (acquired from Annual Energy Outlook 2021[16] for the year 2020) and the specific emission values by fuel types. We used average U.S. electricity mix (for the year 2020) as the source of indirect (upstream) electricity.

The life-cycle GHG emission values and sources are provided in Table S5. Moreover, uncertainty analysis for the life-cycle GHG emissions captures a ±10% variation for the amounts of chemicals, materials, and fuels, as well as the available range of data for bioCNG, PHB, and SCP credit. The details for uncertainty analysis are provided in Table S6.

Table S1. Input parameters used for techno-economic modeling and single-point sensitivity analysis.

Parameters	Unit	Baseline	Minimum	Maximum
Corn stover composition[1]				
Moisture	wt.%	20		
Glucan	wt.% (dry matter)	35.5		
Xylan	wt.% (dry matter)	25.3		
Lignin	wt.% (dry matter)	16.2		
Proteins	wt.% (dry matter)	3.7		
Soluble sugars	wt.% (dry matter)	10.53		
Acetate	wt.% (dry matter)	2.2		
Other solids	wt.% (dry matter)	6.53		
Feedstock supply and handling[1]				
*Corn stover supply cost	$/dry t	100.2	-25%	+25%
DMR pretreatment[2,17–19]				
Pretreatment time	hour	2		
Pretreatment temperature	°C	80		
Solid loading rate	wt.%	30		
*NaOH loading rate	kg/t dry biomass	50	50	70
*Solubilized xylan	wt.%	10	10	20
*Solubilized glucan	wt.%	2	0	2
*Solubilized lignin	wt.%	30	30	60
*Refining energy consumption	kWh/t dry biomass	200	100	400
NaOH price	$/kg	0.40		
Enzymatic hydrolysis and fermentation[9,20,21]				
*Solid loading rate	wt.%	25	20	30
*Enzyme loading rate	mg/g glucan	20	10	20
Enzyme price	$/kg	4.29		
*Glucan to glucose	wt.%	90	80	95
*Xylan to xylose	wt.%	90	80	95
Hydrolysis time	hour	72		
*Glucose conversion	wt.%	95	85	95
*Xylose conversion	wt.%	85	75	85
*Fermentation time	hour	36	24	48
Corn steer liquor price	$/kg	0.06		
Diammonium phosphate price	$/kg	0.97		
Recovery and separation[20]				
Recovery of bioethanol	wt.%	99		
Onsite energy generation				
Electricity price[22]	$/kWh	0.068		
Natural gas price[23]	$/kg	0.13		
Wastewater treatment				
Digester temperature	°C	35		
*Digester residence time[24]	day	25	15	30
*Manure price[25]	$/dry t	60	40	80
*Food waste tipping fee[26]	$/wet t	51.81	41.89	68.34
Hog manure transportation (4,600 t/day)	$/wet t	8.94		
Cattle manure transportation (1,100 t/day)	$/wet t	9.01		
Food waste transportation (400 t/day)	$/wet t	9.29		
Compositions of organic wastes	%	See Table S2		

*CH_4 yields of organic wastes	mL CH_4/g VS	See Table S2		
CH_4 yield of DMR pretreated slurry	mL CH_4/g VS	356		
BioCNG production				
H_2O removal				
*Capacity of silica gel[27]	mg adsorbate/g	400	400	750[28]
Silica gel price[8]	$/kg	2.5		
H_2S removal[6]				
$FeCl_3$ price[29]	$/kg	0.50		
*Capacity of impregnated activated carbon	mg adsorbate/g	100[6]	100[6]	400[30]
Impregnated activated carbon price[6]	$/kg	5		
CO_2 removal[9]				
*Membrane module cost	$/m^2	125	100	150
*CH_4 loss	%	5	0.5[31]	20
Membrane life	year	5		
BioCNG price[32]	$/kg	0.81		
PHB/SCP production				
Reactor temperature[10,11]	°C	38		
*Cell growth reactor residence time[11,33]	hour	48	24	72
Cell density (growth reactor)[11,33]	g/L	20		
PHB concentration (growth reactor)[10]	wt.%	3		
*PHB accumulation reactor residence time[10,11]	hour	48	24	48
Cell density (accumulation reactor)[10]	g/L	50		
PHB concentration (accumulation reactor)[10,11,14,33]	wt.%	60		
*CH_4 utilization efficiency[12]	wt.%	90	80	90
Ammonia price[29]	$/kg	0.45		
KH_2PO_4 price[29]	$/kg	0.80		
Salts ($MgSO_4$) price[29]	$/kg	0.10		
*Total nutrient price			-25%	+25%
PHB recovery[14]	wt.%	90		
PHB purity	wt.%	100		
*PHB price[10]	$/kg	4.75	2.5	7
*SCP price[12]	$/kg	2	1.5	2.5
Economic evaluation[20]				
Daily feedstock processed	dry t/day	2,000		
Annual operating time	hour	8,410		
Plant life	year	30		
Discount rate	%	10		
Total capital investment			-25%	+25%
Total electricity consumption			-25%	+25%
Capital investment for WWT			-25%	+25%
Electricity consumption for WWT			-25%	+25%
Capital investment for bioCNG/PHB/SCP production			-25%	+25%
Electricity consumption for bioCNG/PHB/SCP production			-25%	+25%

*The minimum and maximum values of the input parameters were used to determine the pessimistic and optimistic MESPs (Figure 3).

Table S2. Characteristics of manure and food waste.

	**Average/Baseline**	**Minimum**	**Maximum**
Waste availability in the Corn Belt region for 80-km farm-to-biorefinery distance[25] (wet t/day)			
Hog manure (11% TS)	4,682	191	50,860
Cattle manure (14% TS)	1,138	245	4,519
Food waste (20% TS)	410	20	13,709
Waste composition[a]			
Hog manure			
TS (%)	11	1	31
VS/TS (%)	82	55	90
Lipid/VS (%)	13	0	24
Protein/VS (%)	15	4	29
Carbohydrate/VS (%)	42	0	82
Cattle manure			
TS (%)	14	4	39
VS/TS (%)	80	42	92
Lipid/VS (%)	6	0	10
Protein/VS (%)	18	5	39
Carbohydrate/VS (%)	60	30	77
Food waste			
TS (%)	20	4	64
VS/TS (%)	87	45	100
Lipid/VS (%)	12	0	70
Protein/VS (%)	20	2	64
Carbohydrate/VS (%)	63	4	98
Methane yield (mL CH_4/g VS)[b]			
Hog manure	278	130	425
Cattle manure	164	51	350
Food waste	343	180	541

[a] Data were summarized from previous studies.[34–47]
[b] Data were summarized from previous studies.[34–36,38,41–51]

Table S3. Economic assumptions for determining total capital investment, adopted from Humbird et al.'s study.[20]

Capital Cost Items	Equations/Values/Explanations
Total direct costs (TDC)	Total installed equipment cost + Warehouse + Site development + Additional piping
Total installed equipment cost	Total purchased cost × installation factor (1.7)
Inside-battery-limits (ISBL) equipment costs	Installed equipment costs for primary process areas, including pretreatment, hydrolysis and fermentation, ethanol recovery and separation, as well as bioCNG/PHB/SCP production
Warehouse	4.0% of ISBL
Site development	9.0% of ISBL
Additional piping	4.5% of ISBL
Total indirect costs (TIC)	Prorateable expenses + Field expenses + Home office & construction fee + Project contingency + Other costs
Prorateable expenses	10.0% of TDC
Field expenses	10.0% of TDC
Home office & construction fee	20.0% of TDC
Project contingency	10.0% of TDC
Other costs (start-up, permits, etc.)	10.0% of TDC
Fixed capital investment (FCI)	TDC + TIC
Land	132 acres × $14,000/acre
Working capital	5.0% of FCI
Total capital investment (TCI)	FCI + Land + Working capital

Table S4. Estimates for total fixed operating costs.

Position	2020 salary ($)[a]	No. required for baseline and codigestion scenarios[b]	No. required for codigestion-bioCNG, -PHB, -SCP scenarios[c]
**Labor**			
Plant manager	188,348	1	1
Plant engineer	89,690	2	2
Maintenance supervisor	73,033	1	1
Maintenance technician	51,251	12	16
Lab manager	71,752	1	1
Lab technician	51,251	2	2
Lab tech-enzyme	51,251	2	2
Shift supervisor	61,501	4	5
Shift operators	51,251	20	25
Shift oper-enzyme	51,251	8	8
Yard employees	35,876	4	5
Clerks & secretaries	46,126	3	4
Total salaries		$ 3,295,454	$ 3,900,219
Labor burden (90%)	Total salaries × 90%		
**Overhead**			
Maintenance	3.0% of ISBL		
Property insurance	0.7% of FCI		
**Total fixed operating costs**	Labor (Total salaries + Labor burden) + Overhead (Maintenance + Property insurance)		

[a] Salaries were estimated for rural regions of the U.S. Midwest. Labor indices from the U.S. Bureau of Labor Statistics[52] were used to adjust the salary for 2020 (index: 26.01) based on the salary quote for 2009 (index: 20.30) in Humbird et al.'s study.[20]

[b] Adopted from Humbird et al.'s study.[20]

[c] The number of additional employees for the areas of bioCNG/PHB/SCP production was estimated to be approximately equivalent to that needed for the area of ethanol recovery (i.e., the difference in the number of employees between NREL sugar and ethanol models[20,53]).

Table S5. Life-cycle GHG emission factors for each unit product/process involved in the investigated scenarios.

	Units	GHG Emission Factor (kg CO_{2e}/unit)	References (Notes)
*Corn stover farming & transportation	kg (dry)	0.034	GREET 2021[54]
*NaOH	kg	2.02	GREET 2021[54]
*Cellulase	kg	2.23	GREET 2021[54]
*Corn steep liquor	kg	1.61	GREET 2021[54]
*Diammonium phosphate	kg	1.62	GREET 2021[54]
Inoculum	kg	0.00097	Estimated by developing a process model for inoculum production
*Glucose	kg	0.78	GREET 2021[54]
*Lime, $Ca(OH)_2$	kg	1.28	GREET 2021[54]
*KH_2PO_4	kg	2.19	GREET 2021[54]
*Ammonia	kg	2.53	GREET 2021[54]
*$MgSO_4$	kg	0.12	GREET 2021[54]
*$FeCl_3$	kg	1.58	GREET 2021[54]
Activated carbon	kg	6.6	[55]
Silica gel	kg	1.66	Ecoinvent 3.6[56] (used the value for 'activated silica')
*Diesel	MJ	0.086	GREET 2021[54] (for transportation, including 0.074 kg CO_{2e}/MJ diesel combustion emission)
*Natural gas	MJ	0.068	GREET 2021[54] (including 0.056 kg CO_{2e}/MJ natural gas combustion emission)
*Electricity (U.S. average)	kWh	0.44	GREET 2021[54] (0.4% residual oil, 39.6 % natural gas, 20.0% coal, 20.4% nuclear power, 19.7 % renewables); used for indirect (upstream) electricity
Electricity (petroleum)	kWh	1.08	GREET 2021[54]; used for direct electricity
Electricity (coal)	kWh	0.99	NREL LCA Harmonization[57] (used the median values of summarized publications); used for direct electricity
Electricity (natural gas)	kWh	0.48	
Electricity (nuclear)	kWh	0.013	
Electricity (hydropower)	kWh	0.0066	
Electricity (biopower)	kWh	0.041	
Electricity (solar photovoltaic)	kWh	0.048	
Electricity (wind)	kWh	0.012	
Displaced manure direct land application	kg (wet)	-0.20	[58,59] (including manure storage and land application of manure; inorganic fertilizer displacement was considered assuming 0.0013 kg N/kg wet manure to land)
Displaced food waste landfilling	kg (wet)	-0.54	WARM[60] (used the value for 'food waste')
*Displaced fossil polymer production	kg	-2.02	[54](the median value among general-purpose polystyrene, high-density polyethylene, low-density polyethylene, and polypropylene)
*Displaced fishmeal production	kg	-1.97	Ecoinvent 3.6[56] (used the inputs data for 'fishmeal, 63-65% protein, production from fish residues, region RoW')

*The values were computed using the physical-unit based input-output (IO) matrix (in the SI B).

Table S6. Uncertainty analysis for life-cycle GHG emissions (represented by the uncertainty bars in Figure 5a)

**Unit Product/Process**	**Uncertainty Analysis**
Electricity use or credit	±10% variations of the quantities of consumed or extra electricity
Materials and chemicals	±10% variations of the quantities
Organic waste credits (displacing direct manure land application and food waste landfilling)	No variation on the quantities of hog/cattle manures, food waste, and diesel (used for the transportation to biorefinery).
BioCNG credit	No variation on the bioCNG quantity. Credited for offsetting fossil natural gas (0.068 kg CO_{2e}/MJ[54]) or diesel fuel (0.086 kg CO_{2e}/MJ[54]). The CO_2 emission from BioCNG combustion is considered to be biogenic, which is offset by the CO_2 sequestered during the photosynthesis of plants (the origins for organic wastes).
PHB credit	No variation on the PHB quantity. Credited for offsetting fossil-based polymers: 1.60 kg CO_{2e}/kg polypropylene (minimum), 2.02 kg CO_{2e}/kg polyethylene (baseline), and 2.92 kg CO_{2e}/kg general-purpose polystyrene (maximum).[54]
SCP credit	No variation on the SCP quantity. Credited for offsetting fishmeal (1.97 kg CO_{2e}/kg fishmeal[56,61]) or soybean meal (0.46 kg CO_{2e}/kg soybean meal[54]). Credits were determined by normalizing to the protein content of each product (72% protein for SCP,[12] 65% for fishmeal,[56] and 46% for soybean meal[62]).

Table S7. The numeric values used to plot Figure 3.

**Process areas and credits**	**Cost contribution for different scenarios** ($/gasoline gallon equivalent)				
	**S1**	**S2**	**S3**	**S4**	**S5**
Feedstock supply & handling	1.91	1.91	1.91	1.92	1.92
Pretreatment	1.27	1.18	1.29	1.36	1.36
Hydrolysis & fermentation	0.15	0.15	0.15	0.15	0.15
Recovery & separation	0.13	0.11	0.13	0.14	0.14
WWT	0.88	1.35	1.40	1.78	1.43
Onsite energy generation	0.39	0.56	0.40	0.44	0.48
Utilities	0.08	0.07	0.09	0.12	0.10
Manure delivered cost	/	0.79	0.79	0.79	0.79
BioCNG production	/	/	0.13	/	/
PHB production	/	/	/	1.16	/
SCP production	/	/	/	/	0.99
Electricity credit	/	-0.29	/	/	/
Food waste tipping revenue	/	-0.14	-0.14	-0.14	-0.14
BioCNG revenue	/	/	-1.51	/	/
PHB revenue	/	/	/	-3.06	/
SCP revenue	/	/	/	/	-2.14
**MESP**	4.81	5.69	4.65	4.66	5.09
Uncertainty bar (-)	1.14	1.76	2.04	4.22	2.93
Uncertainty bar (+)	2.52	3.15	3.27	4.85	3.87

Table S8. The numeric values used to plot Figure 4. The quantities of organic wastes vary in S4 and S4-FW, a decrease by half and an increase by 50% relative to average resource availability (X, a total of 6,100 wet metric ton/day) were modeled for comparison.

**Process areas and credits**	**Cost contribution for different scenarios** ($/gasoline gallon equivalent)								
	S1	S2	S4			S2-FW	S4-FW		
		X	0.5X	X	1.5X	X	0.5X	X	1.5X
Feedstock supply & handling	1.91	1.91	1.92	1.92	1.92	1.92	1.92	1.93	1.93
Pretreatment	1.27	1.18	1.35	1.36	1.35	1.19	1.37	1.38	1.37
Hydrolysis & fermentation	0.15	0.15	0.15	0.15	0.15	0.15	0.16	0.16	0.16
Recovery & separation	0.13	0.11	0.14	0.14	0.14	0.11	0.14	0.14	0.14
Wastewater treatment	0.88	1.35	1.39	1.78	2.09	1.39	1.50	2.10	2.51
Onsite energy generation	0.39	0.56	0.41	0.44	0.49	0.66	0.42	0.47	0.54
Utilities	0.08	0.07	0.10	0.12	0.12	0.07	0.11	0.14	0.16
Manure delivered cost	/	0.79	0.40	0.79	1.19	/	/	/	/
PHB production	/	/	0.68	1.16	1.41	/	1.07	1.89	2.48
Electricity credit	/	-0.29	/	/	/	-0.61	/	/	/
Food waste tipping revenue	/	-0.14	-0.07	-0.14	-0.22	-2.19	-1.10	-2.19	-3.29
PHB revenue	/	/	-1.73	-3.06	-3.81	/	-2.78	-4.96	-6.68
**MESP**	4.81	5.69	4.73	4.66	4.83	2.69	2.81	1.04	-0.68
PHB selling price sensitivity (- and +)	/	/	0.82	1.45	1.80	/	1.31	2.35	3.16

Table S9. The numeric values used to plot Figure 5a.

Process areas and credits	Life-cycle GHG emissions for different scenarios (gCO_{2e} per MJ of ethanol)				
	S1	S2	S3	S4	S5
Feedstock production & transportation	4.39	4.39	4.39	4.39	4.39
Materials & chemicals	19.58	19.84	20.00	26.29	24.71
Electricity use	9.81	0.00	15.35	47.46	43.07
Electricity credit	/	-18.75	/	/	/
Manure credit	/	-70.21	-70.21	-70.21	-70.21
Food waste credit	/	-13.90	-13.90	-13.90	-13.90
BioCNG credit	/	/	-52.86	/	/
PHB credit	/	/	/	-10.01	/
SCP credit	/	/	/	/	-17.92
**Total**	33.78	-78.62	-97.23	-15.97	-29.86
Uncertainty bar (-)	2.94	3.86	3.16	11.82	6.78
Uncertainty bar (+)	2.94	3.86	11.14	9.50	18.84

Table S10. Life-cycle fossil energy demand (including petroleum, natural gas, and coal) for each unit product/process involved in the investigated scenarios.

	Units	**Impact Factor** (MJ/unit)			**References (Notes)**
		Petroleum	Natural gas	Coal	
Corn stover farming & transportation	kg (dry)	0.51	0.36	0.033	GREET 2021[54]
Cellulase	kg	2.72	20.07	3.14	GREET 2021[54]
NaOH	kg	1.17	21.72	5.65	GREET 2021[54]
Corn steep liquor	kg	3.54	9.09	0.49	GREET 2021[54]
Diammonium phosphate	kg	5.25	17.39	1.74	GREET 2021[54]
Glucose	kg	1.21	6.63	0.33	GREET 2021[54]
Lime, $Ca(OH)_2$	kg	0.20	0.40	3.09	GREET 2021[54]
KH_2PO_4	kg	6.33	13.79	4.65	GREET 2021[54]
Ammonia	kg	1.22	40.47	0.33	GREET 2021[54]
$MgSO_4$	kg	0.0052	1.40	0.0014	GREET 2021[54]
$FeCl_3$	kg	0.29	10.86	8.72	GREET 2021[54]
Diesel	MJ	1.04	0.078	0.0026	GREET 2021[54]
Natural gas	MJ	0.0040	1.10	0.0011	GREET 2021[54]
Electricity (U.S. average)	kWh	0.096	3.45	2.20	GREET 2021[54] (0.4% residual oil, 39.6 % natural gas, 20.0% coal, 20.4% nuclear power, 19.7 % renewables)
Displaced manure direct land application	kg (wet)	0.012	0.0009	0	[58,59] (including manure storage and land application of manure; inorganic fertilizer displacement was considered assuming 0.0013 kg N/kg wet manure to land)
Displaced food waste landfilling	kg (wet)	0.21	0.016	0.0005	WARM[60] (used the value for 'food waste')
Displaced fossil polymer production	kg	29.24	39.88	1.12	[54](the median value among general-purpose polystyrene, high-density polyethylene, low-density polyethylene, and polypropylene)
Displaced fishmeal production	kg	23.31	10.30	0.29	Ecoinvent 3.6[56] (used the inputs data for 'fishmeal, 63-65% protein, production from fish residues, region RoW')

Table S11. Uncertainty analysis for life-cycle fossil energy demand (represented by the uncertainty bars in Figure S4)

Unit Product/Process	Uncertainty Analysis
Electricity use or credit	±10% variations of the quantities of consumed or extra electricity
Materials and chemicals	±10% variations of the quantities
Organic waste credits (displacing direct manure land application and food waste landfilling)	No variation on the quantities of hog/cattle manures, food waste, and diesel (used for the transportation to biorefinery).
BioCNG credit	No variation on the bioCNG quantity. Credited for offsetting fossil natural gas (0.0040, 1.10, and 0.0011 MJ/MJ for petroleum, natural gas, and coal[54]) or diesel fuel (1.04, 0.078, and 0.0026 MJ/MJ for petroleum, natural gas, and coal[54]).
PHB credit	No variation on the PHB quantity. Credited for offsetting fossil-based polymers: the respective life-cycle petroleum, natural gas, and coal demands are 43.58, 16.35, and 0.72 MJ/kg for polypropylene (minimum); 29.24, 39.88, and 1.12 MJ/kg for polyethylene (baseline); 33.04, 49.14, and 1.33 MJ/kg for general-purpose polystyrene (maximum).[54]
SCP credit	No variation on the SCP quantity. Credited for offsetting fishmeal (21.04, 9.30, and 0.26 MJ/kg for petroleum, natural gas, and coal, respectively[56,61]) or soybean meal (1.02, 1.73, and 0.65 for petroleum, natural gas, and coal, respectively[54]). Credits were determined by normalizing to the protein content of each product (72% protein for SCP,[12] 65% for fishmeal,[56] and 46% for soybean meal[62]).

Provided in the separately attached excel file are three additional tables:
Table S12. Notes & References: Notes for products/inputs (e.g., definition of abbreviations) and the data sources.
Table S13. 'I-O Table': Rows represent products and columns indicate inputs. The unit of each input is included at the end of input name.
Table S14. 'Impact Vectors': Rows represent products/inputs and columns indicate impact vectors used in GHG calculation.

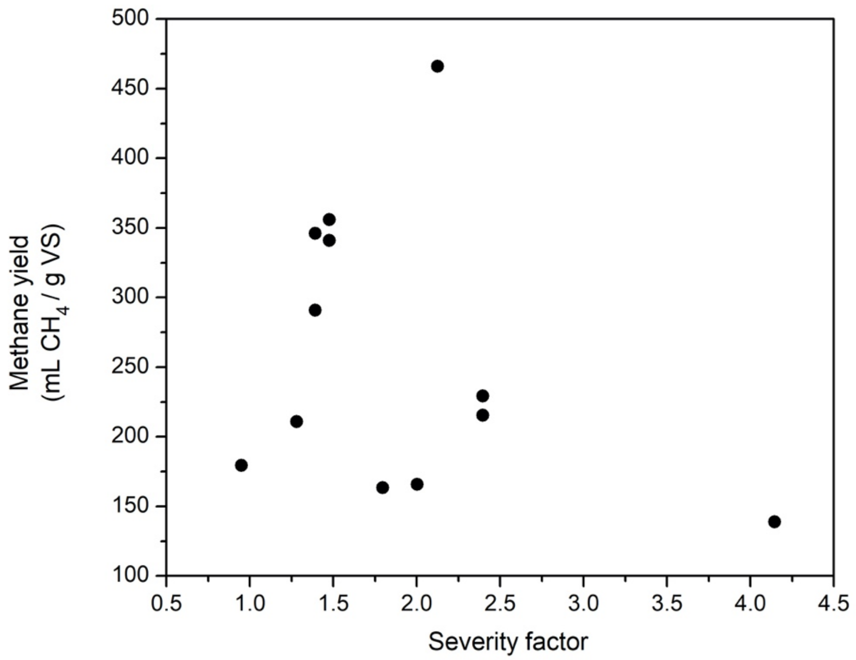

Figure S1. Reported methane yield (mL CH_4/g VS) as a function of the severity factor for alkaline pretreatment of lignocellulosic feedstocks with NaOH. The severity factor herein is a relationship between pretreatment temperature and time. The equation to calculate the severity factor is $Severity\ Factor = \ log_{10}(t \times \exp\left[\frac{T-100}{14.75}\right])$, where t is the reaction time (min) and T is the pretreatment temperature (°C).[63,64] The data were collected from previous studies.[65–72] Our model assigns a methane yield of 356 mL CH_4/g VS (i.e., the maximum value at the severity factor around 1.5 used for the DMR process in our study) to the waste streams that are generated from ethanol production and delivered to the AD facility.

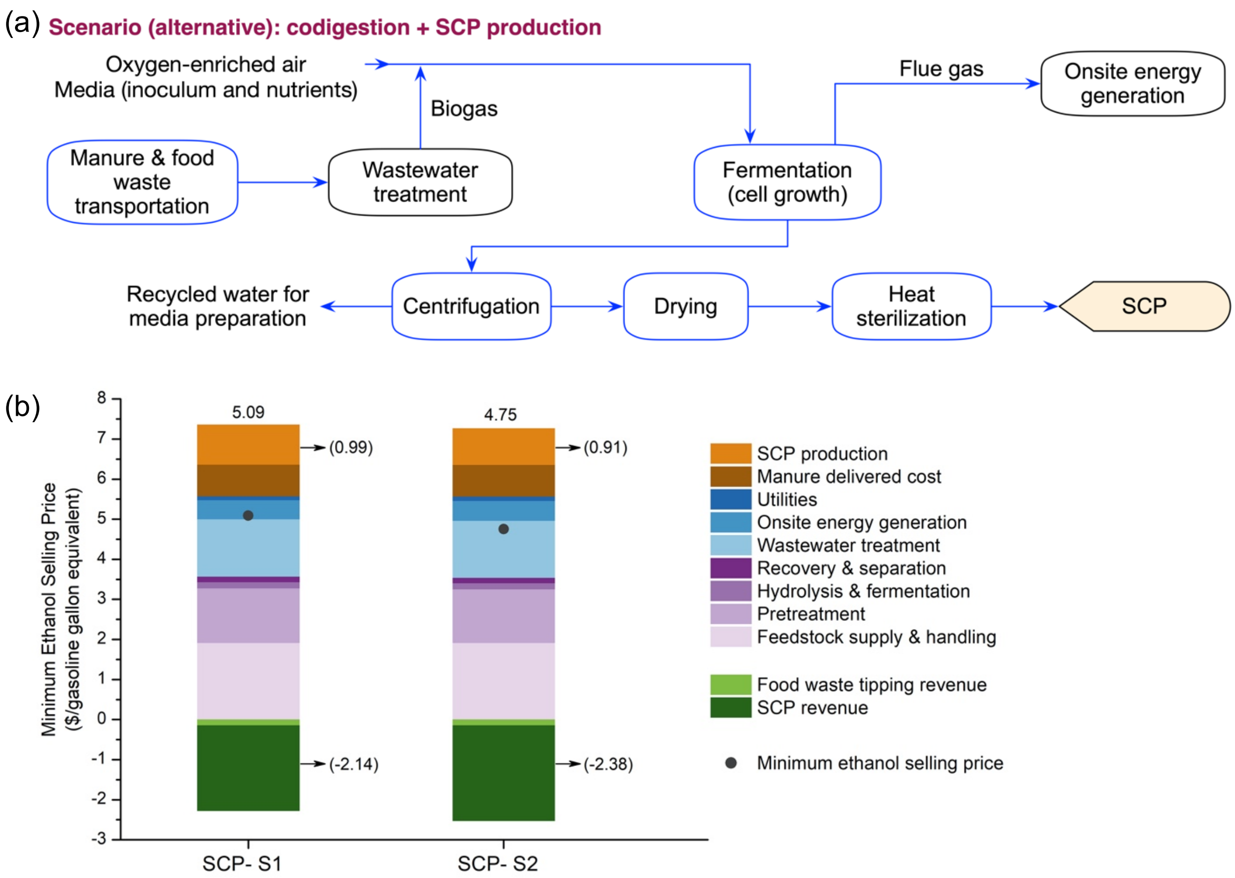

Figure S2. One alternative SCP production scenario was investigated, where cells are harvested from the cell growth reactor to produce SCP, different from the scenario shown in Figure 2d where cells containing PHB are harvested from the PHB accumulation reactor. (a) Process flow diagram for this alternative SCP production scenario. (b) Comparison of minimum ethanol selling price (MESP, $/gasoline gallon equivalent) between two SCP production scenarios; SCP-S1 refers to the scenario using PHB-containing cells (Figure 2d) while SCP-S2 refers to that using gross cells (a). Another main difference related to the input parameter between these two scenarios is the cell density, of which cell growth reactor is increased to 50 g/L in SCP-S2 compared to 20 g/L in SCP-S1, while the cell density of PHB accumulation reactor is 50 g/L in SCP-S1. The amounts of organic wastes for codigestion are 4,600, 1,100, and 400 wet metric ton/day for hog manure, cattle manure, and food waste, respectively. The contribution to the MESP is shown by process areas and credits (food waste tipping revenue and SCP selling revenue). The MESP values (labeled on top of each bar) were determined using the baseline values for input parameters. The values in the parentheses represent the cost contributions to MESP from SCP production and SCP selling revenue, respectively.

(a) Scenario: biogas onsite combustion

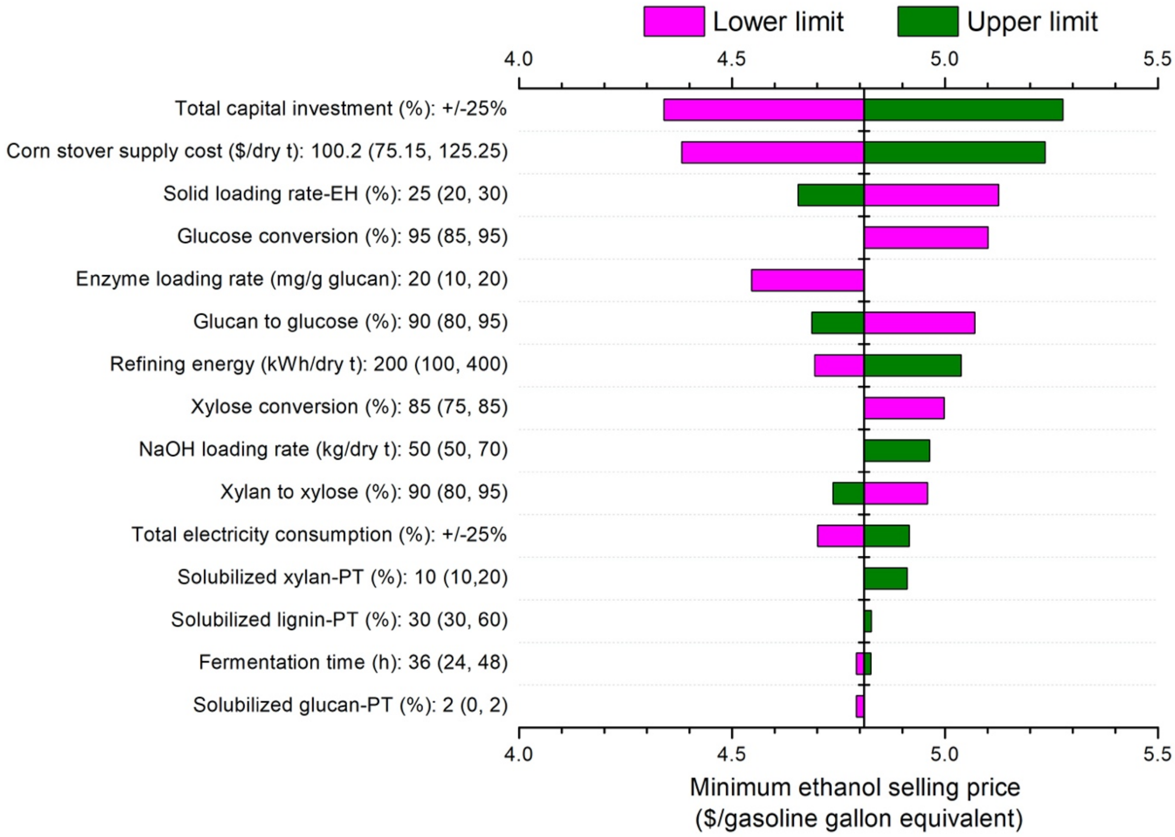

(b) Scenario: codigestion + combustion

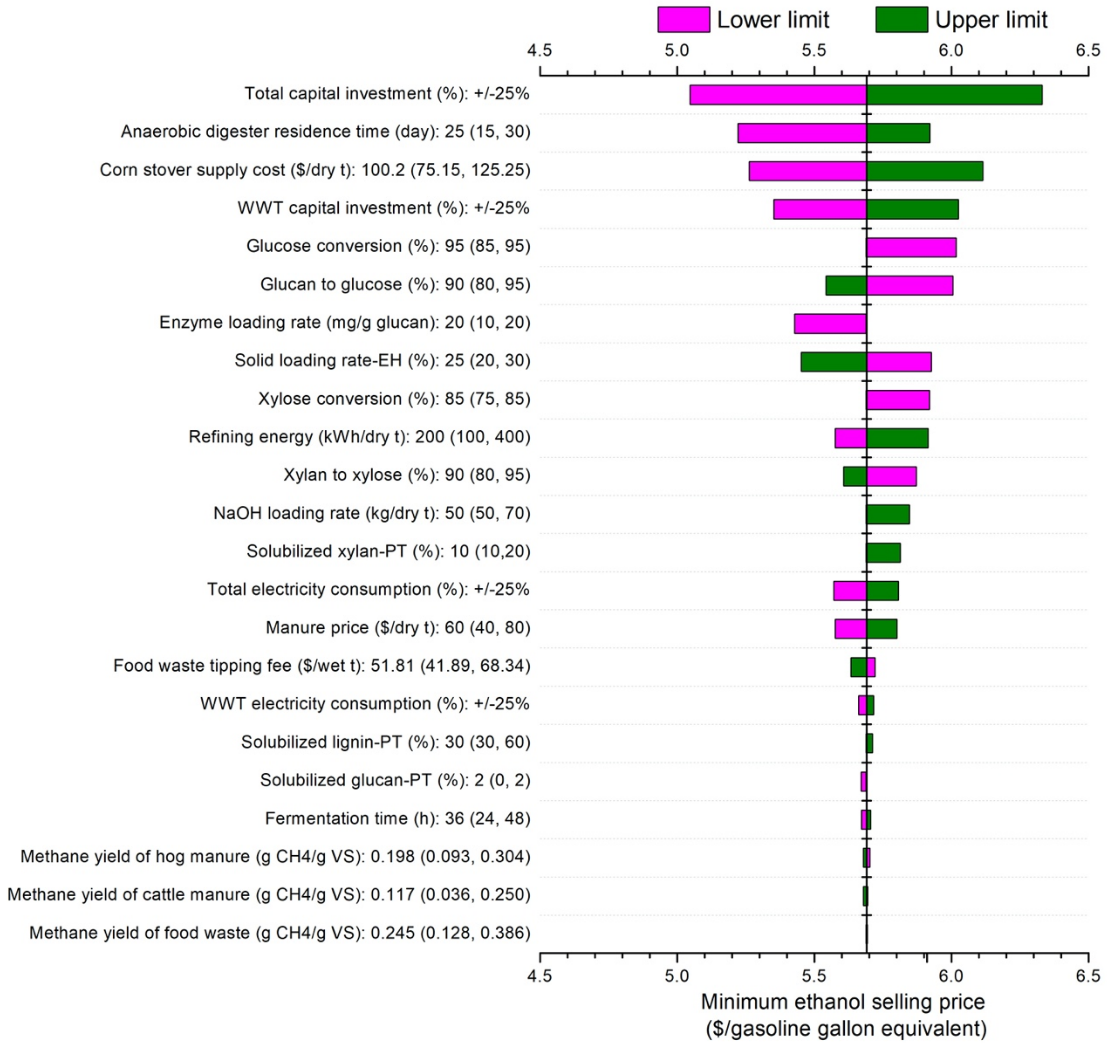

(c) Scenario: codigestion + bioCNG production

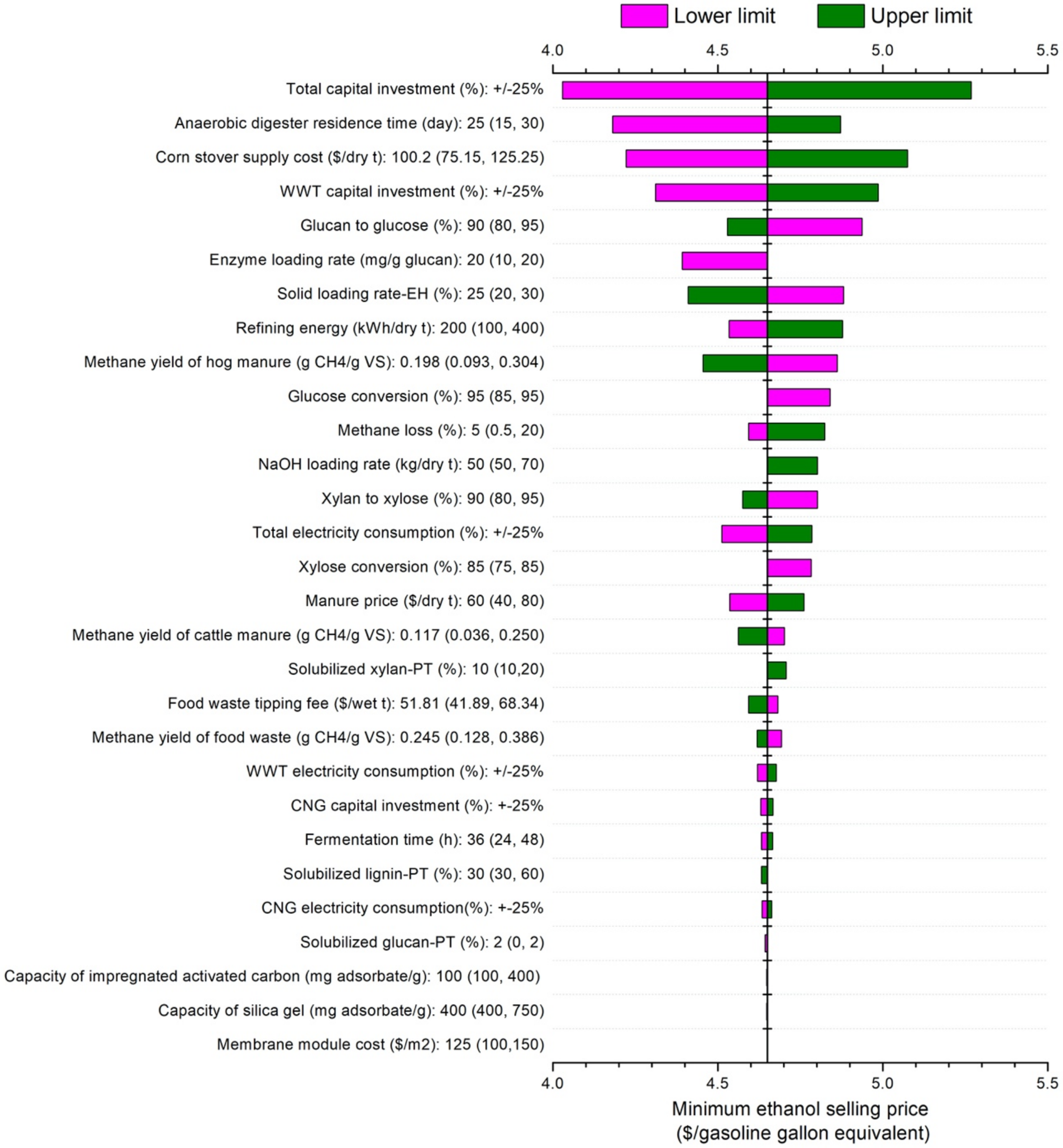

(d) Scenario: codigestion + PHB production

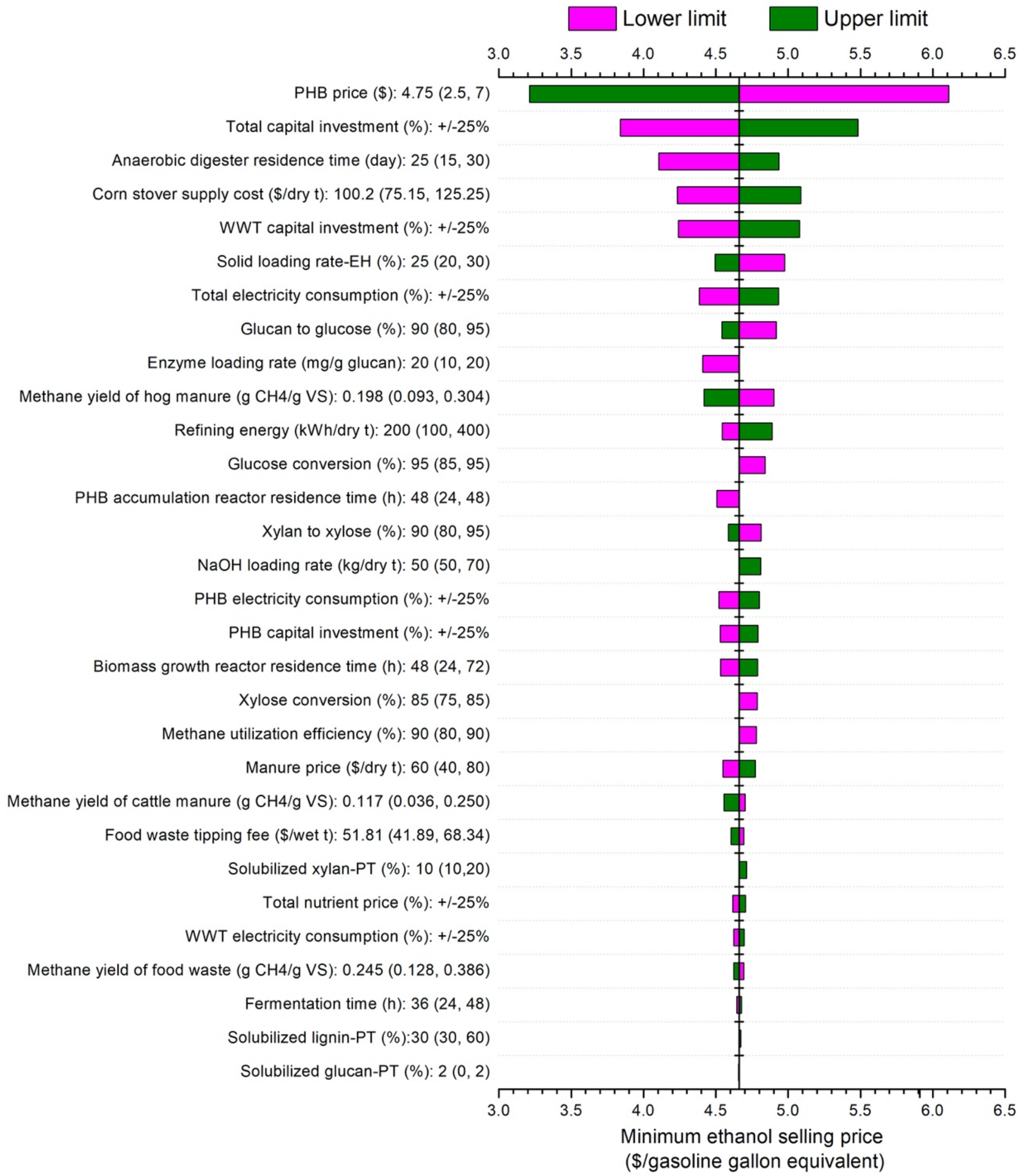

(e) Scenario: codigestion + SCP production

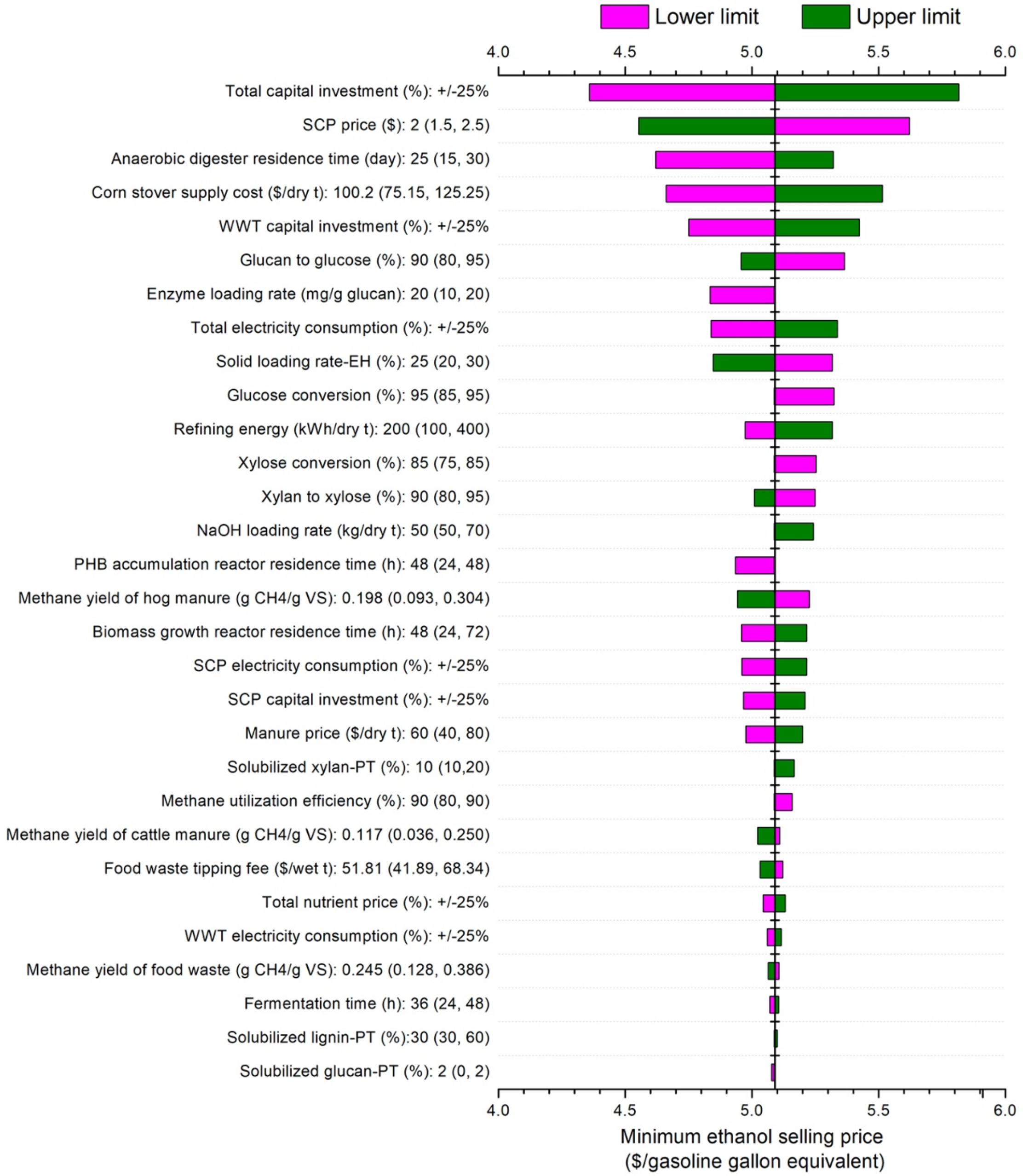

Figure S3. Single point sensitivity analyses to identify the most influential input parameters to minimum ethanol selling price for five scenarios, including (a) biorefinery with biogas onsite combustion, (b) integrated biorefinery with codigestion of organic wastes and biogas onsite combustion, (c) integrated biorefinery with codigestion of organic wastes and biogas upgrading to bioCNG, (d) integrated biorefinery with codigestion of organic wastes and biogas conversion to PHB, and (e) integrated biorefinery with codigestion of organic wastes and biogas conversion to SCP. The amounts of organic wastes for codigestion are 4,600, 1,100, and 400 wet metric ton/day for hog manure, cattle manure, and food waste, respectively. (PT: pretreatment; EH: enzymatic hydrolysis; VS: volatile solid)

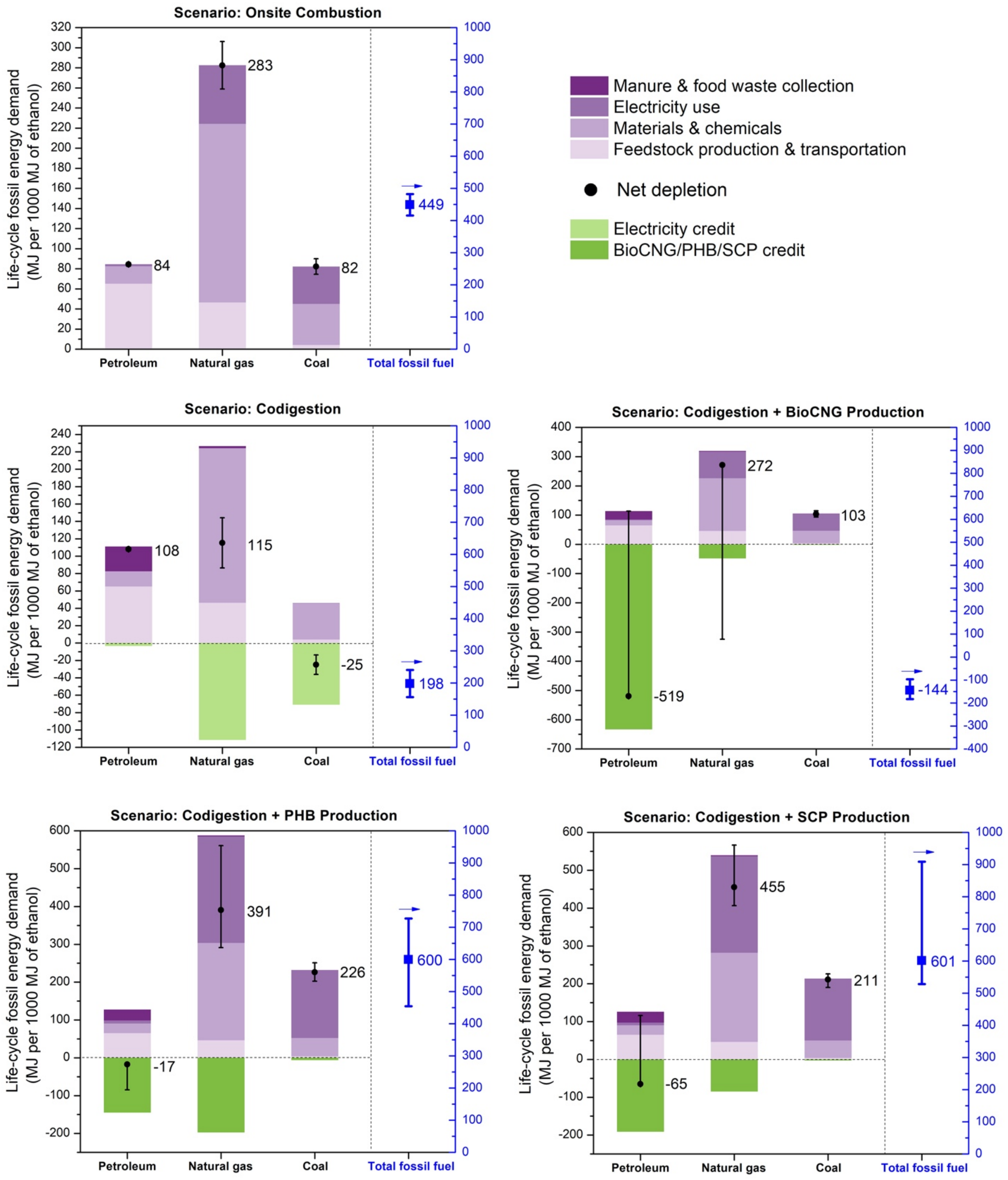

Figure S4. Life-cycle fossil energy demand per 1,000 MJ of ethanol produced for different scenarios. The contribution to crude oil, natural gas, and coal is shown by input categories and offset credits (stacked bar charts referred to left y-axis). The total fossil fuel demand is shown by

blue square (referred to right y-axis). The presented scenarios include the baseline biorefinery (onsite combustion) and the integrated biorefineries with codigestion of organic wastes and biogas utilization routes to bioCNG, PHB, and SCP, respectively. The amounts of organic wastes for codigestion are 4,600, 1,100, and 400 wet metric ton/day for hog manure, cattle manure, and food waste, respectively. The fossil energy demand for each scenario was determined using the baseline values (Table S10) for inputs and offset credits (bioCNG for offsetting diesel herein). Uncertainty bars capture variations on all inputs and offset credits (summarized in Table S11). For biogas-to-bioCNG scenario, the high uncertainty arises from bioCNG offset options that alter from diesel to natural gas. Note that for the fossil energy demand results, both direct and indirect (upstream) electricity sources are average U.S. electricity mix. Results show that codigestion of organic wastes followed by biogas combustion significantly decreases the demand for natural gas and coal due to the generation of excess electricity compared to the baseline scenario. A minimum and negative fossil energy demand is reached upon upgrading biogas to bioCNG that brings considerable credit from offsetting diesel. Although using biogas for PHB production to displace fossil polymers leads to a notable offset credit on petroleum depletion, high electricity consumption in the scenarios of PHB and SCP production significantly increases the amounts of natural gas and coal, resulting in the highest fossil energy use out of all scenarios.

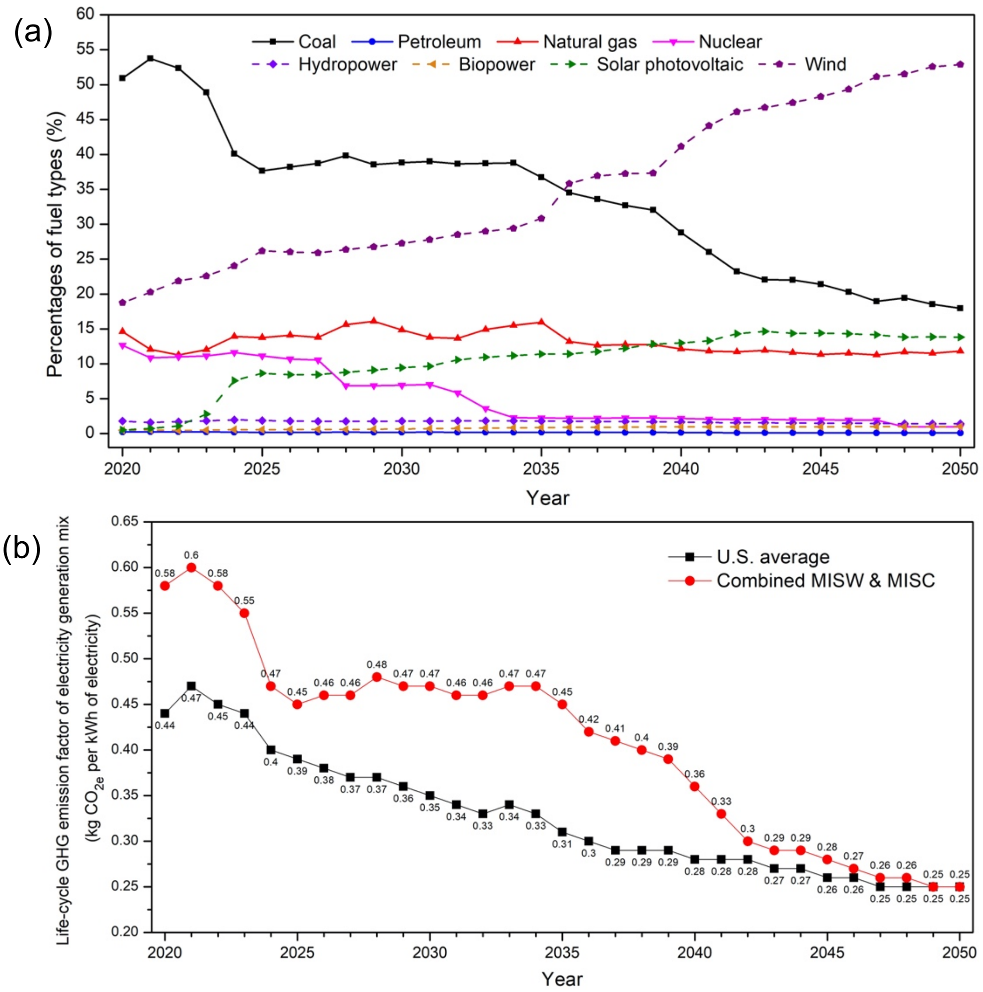

Figure S5. (a) Electric power projections (2020 to 2050) by fuel types for two electricity subregions, midcontinent independent system operator west (MISW) and central (MISC). The data were taken from the Annual Energy Outlook 2021 & 2022 considering the Low Renewable Cost case (a high renewable energy penetration scenario). (b) The life-cycle GHG emission values per unit of electricity generated for average U.S. electricity mix and combined MISW and MISC regions, respectively.

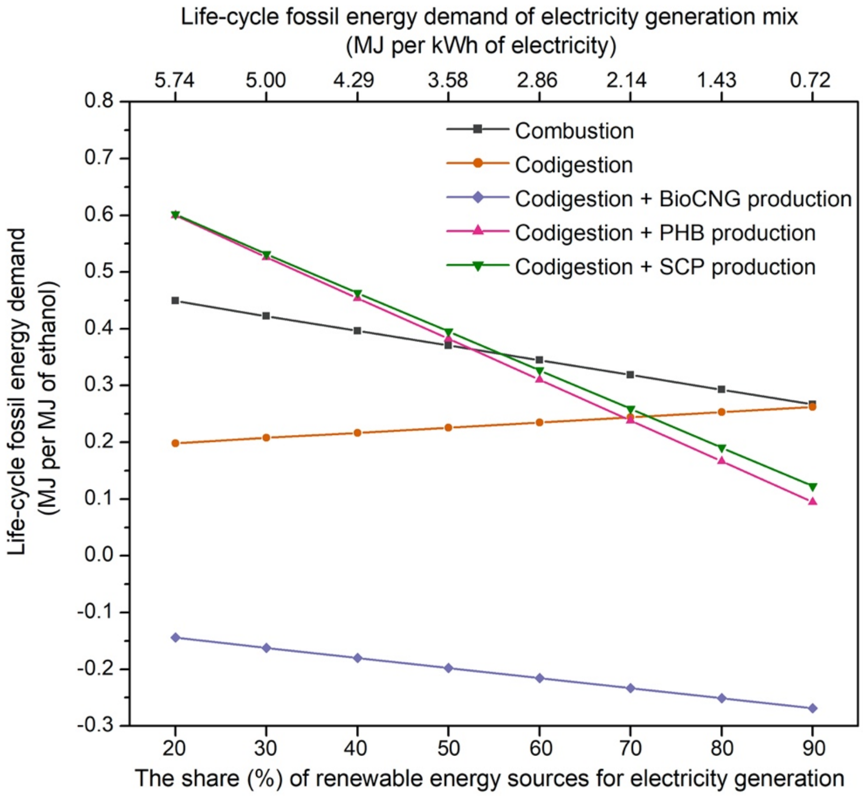

Figure S6. Change in the life-cycle fossil energy demand as a function of the increasing share of renewable electricity while the corresponding life-cycle fossil energy demand per unit of electricity generated is indicated by top x-axis. The starting data points refer to the results in Figure S4. The presented scenarios include the baseline biorefinery (onsite combustion) and the integrated biorefineries with codigestion of organic wastes and biogas utilization routes to bioCNG, PHB, and SCP, respectively. Note that for the fossil energy demand results, both direct and indirect (upstream) electricity sources are assumed to be average U.S. electricity mix.

References

(1) Baral, N. R.; Davis, R.; Bradley, T. H. Supply and value chain analysis of mixed biomass feedstock supply system for lignocellulosic sugar production. *Biofuels, Bioprod. Bioref.* **2019**, *13*, 635–659.

(2) Chen, X.; Kuhn, E.; Jennings, E. W.; Nelson, R.; Tao, L.; Zhang, M.; Tucker, M. P. DMR (deacetylation and mechanical refining) processing of corn stover achieves high monomeric sugar concentrations (230 g L^{-1}) during enzymatic hydrolysis and high ethanol concentrations (>10% v/v) during fermentation without hydrolysate purification or concentration. *Energy Environ. Sci.* **2016**, *9*, 1237–1245.

(3) Rodrigues, R. P.; Rodrigues, D. P.; Klepacz-Smolka, A.; Martins, R. C.; Quina, M. J. Comparative analysis of methods and models for predicting biochemical methane potential of various organic substrates. *Sci. Total Environ.* **2019**, *649*, 1599–1608.

(4) Meegoda, J. N.; Li, B.; Patel, K.; Wang, L. B. A review of the processes, parameters, and optimization of anaerobic digestion. *Int. J. Environ. Res. Public Health* **2018**, *15*, Article 2224.

(5) Chen, X. Y.; Vinh-Thang, H.; Ramirez, A. A.; Rodrigue, D.; Kaliaguine, S. Membrane gas separation technologies for biogas upgrading. *RSC Adv.* **2015**, *5*, 24399–24448.

(6) Chaemchuen, S.; Zhou, K.; Verpoort, F. From biogas to biofuel: materials used for biogas cleaning to biomethane. *ChemBioEng Reviews* **2016**, *3*, 250–265.

(7) Beil, M.; Beyrich, W. Biogas upgrading to biomethane. In *The Biogas Handbook*; Elsevier, 2013; pp. 342–377.

(8) Grande, C. A.; Morence, D. G. B.; Bouzga, A. M.; Andreassen, K. A. Silica gel as a selective adsorbent for biogas drying and upgrading. *Ind Eng Chem Res* **2020**, *59*, 10142–10149.

(9) Yang, M.; Baral, N. R.; Anastasopoulou, A.; Breunig, H. M.; Scown, C. D. Cost and life-cycle greenhouse gas implications of integrating biogas upgrading and carbon capture technologies in cellulosic biorefineries. *Environ. Sci. Technol.* **2020**, *54*, 12810–12819.

(10) Levett, I.; Birkett, G.; Davies, N.; Bell, A.; Langford, A.; Laycock, B.; Lant, P.; Pratt, S. Techno-economic assessment of poly-3-hydroxybutyrate (PHB) production from methane—The case for thermophilic bioprocessing. *Journal of Environmental Chemical Engineering* **2016**, *4*, 3724–3733.

(11) Helm, J.; Wendlandt, K. D.; Jechorek, M.; Stottmeister, U. Potassium deficiency results in accumulation of ultra-high molecular weight poly-beta-hydroxybutyrate in a methane-utilizing mixed culture. *J. Appl. Microbiol.* **2008**, *105*, 1054–1061.

(12) Pikaar, I.; Matassa, S.; Bodirsky, B. L.; Weindl, I.; Humpenöder, F.; Rabaey, K.; Boon, N.; Bruschi, M.; Yuan, Z.; van Zanten, H.; et al. Decoupling livestock from land use through industrial feed production pathways. *Environ. Sci. Technol.* **2018**, *52*, 7351–7359.

(13) Ghatnekar, M. S.; Pai, J. S.; Ganesh, M. Production and recovery of poly-3-hydroxybutyrate from Methylobacterium sp V49. *Journal of Chemical Technology & Biotechnology: International Research in Process, Environmental & Clean Technology* **2002**, *77*, 444–448.

(14) Rostkowski, K. H.; Criddle, C. S.; Lepech, M. D. Cradle-to-gate life cycle assessment for a cradle-to-cradle cycle: biogas-to-bioplastic (and back). *Environ. Sci. Technol.* **2012**, *46*, 9822–9829.

(15) Matassa, S.; Boon, N.; Pikaar, I.; Verstraete, W. Microbial protein: future sustainable

food supply route with low environmental footprint. *Microb Biotechnol* **2016**, *9*, 568–575.
(16) U.S. Energy Information Administration. Annual Energy Outlook 2022. https://www.eia.gov/electricity/ (accessed Mar 28, 2022).
(17) Kuhn, E. M.; Chen, X.; Tucker, M. P. Deacetylation and mechanical refining (DMR) and deacetylation and dilute acid (DDA) pretreatment of corn stover, switchgrass, and a 50:50 corn stover/switchgrass blend. *ACS Sustain. Chem. Eng.* **2020**, *8*, 6734–6743.
(18) Chen, X.; Shekiro, J.; Pschorn, T.; Sabourin, M.; Tao, L.; Elander, R.; Park, S.; Jennings, E.; Nelson, R.; Trass, O.; et al. A highly efficient dilute alkali deacetylation and mechanical (disc) refining process for the conversion of renewable biomass to lower cost sugars. *Biotechnol Biofuels* **2014**, *7*, 98.
(19) Chen, X.; Wang, W.; Ciesielski, P.; Trass, O.; Park, S.; Tao, L.; Tucker, M. P. Improving sugar yields and reducing enzyme loadings in the deacetylation and mechanical refining (DMR) process through multistage disk and szego refining and corresponding techno-economic analysis. *ACS Sustain. Chem. Eng.* **2016**, *4*, 324–333.
(20) Humbird, D.; Davis, R.; Tao, L.; Kinchin, C.; Hsu, D.; Aden, A.; Schoen, P.; Lukas, J.; Olthof, B.; Worley, M.; et al. *Process Design and Economics for Biochemical Conversion of Lignocellulosic Biomass to Ethanol: Dilute-Acid Pretreatment and Enzymatic Hydrolysis of Corn Stover*; National Renewable Energy Laboratory (NREL): Golden, CO (United States), 2011.
(21) Baral, N. R.; Kavvada, O.; Mendez Perez, D.; Mukhopadhyay, A.; Lee, T. S.; Simmons, B. A.; Scown, C. D. Greenhouse gas footprint, water-intensity, and production cost of bio-based isopentenol as a renewable transportation fuel. *ACS Sustain. Chem. Eng.* **2019**, *7*, 15434–15444.
(22) U.S. Energy Information Administration. Short-term energy outlook. https://www.eia.gov/outlooks/steo/ (accessed Aug 9, 2020).
(23) U.S. Energy Information Administration. Natural gas prices. https://www.eia.gov/naturalgas/ (accessed Aug 9, 2020).
(24) Mir, M. A.; Hussain, A.; Verma, C. Design considerations and operational performance of anaerobic digester: A review. *Cogent Engineering* **2016**, *3*.
(25) Langholtz, M. H.; Stokes, B. J.; Eaton, L. M. *2016 Billion-ton report: Advancing domestic resources for a thriving bioeconomy, Volume 1: Economic availability of feedstock*; Oak Ridge National Laboratory, 2016; pp. 1–411.
(26) Staley, B. F.; Kantner, D. L.; Choi, J. *Analysis of MSW Landfill Tipping Fees 2020*; Environmental Research & Education Foundation, 2021.
(27) SorbentSystems. Desiccant chart comparisons. https://www.sorbentsystems.com/index.html (accessed Nov 9, 2020).
(28) GIEBEL Adsorber. Silica gel features. https://www.giebel-adsorber.de/en/products/adsorbents-silicagel (accessed Nov 8, 2020).
(29) Echemi. Market price and insights. https://www.echemi.com/weekly-price.html (accessed Jul 31, 2020).
(30) Xiao, Y.; Wang, S.; Wu, D.; Yuan, Q. Catalytic oxidation of hydrogen sulfide over unmodified and impregnated activated carbon. *Separation and Purification Technology* **2008**, *59*, 326–332.
(31) Ardolino, F.; Cardamone, G. F.; Parrillo, F.; Arena, U. Biogas-to-biomethane upgrading: A comparative review and assessment in a life cycle perspective. *Renewable and Sustainable Energy Reviews* **2021**, *139*, 110588.

(32) U.S. Department of Energy. Fuel prices. https://afdc.energy.gov/fuels/prices.html (accessed Nov 9, 2020).
(33) Wendlandt, K. D.; Jechorek, M.; Helm, J.; Stottmeister, U. Producing poly-3-hydroxybutyrate with a high molecular mass from methane. *J. Biotechnol.* **2001**, *86*, 127–133.
(34) Mostafa Imeni, S.; Pelaz, L.; Corchado-Lopo, C.; Maria Busquets, A.; Ponsá, S.; Colón, J. Techno-economic assessment of anaerobic co-digestion of livestock manure and cheese whey (Cow, Goat & Sheep) at small to medium dairy farms. *Bioresour. Technol.* **2019**, *291*, Article 121872.
(35) Aui, A.; Li, W.; Wright, M. M. Techno-economic and life cycle analysis of a farm-scale anaerobic digestion plant in Iowa. *Waste Manag.* **2019**, *89*, 154–164.
(36) Imeni, S. M.; Puy, N.; Ovejero, J.; Busquets, A. M.; Bartroli, J.; Pelaz, L.; Ponsá, S.; Colón, J. Techno-economic assessment of anaerobic co-digestion of cattle manure and wheat straw (raw and pre-treated) at small to medium dairy cattle farms. *Waste Biomass Valor.* **2020**, *11*, 4035–4051.
(37) Skaggs, R. L.; Coleman, A. M.; Seiple, T. E.; Milbrandt, A. R. Waste-to-Energy biofuel production potential for selected feedstocks in the conterminous United States. *Renewable and Sustainable Energy Reviews* **2018**, *82*, 2640–2651.
(38) Lehtomäki, A.; Huttunen, S.; Rintala, J. A. Laboratory investigations on co-digestion of energy crops and crop residues with cow manure for methane production: Effect of crop to manure ratio. *Resources, Conservation and Recycling* **2007**, *51*, 591–609.
(39) Lorimor, J.; Powers, W.; Sutton, A. Manure characteristics MWPS-18, Section 1. *Man. Manage. Syst. Ser* **2008**, 1–24.
(40) Li, Y.; Han, Y.; Zhang, Y.; Luo, W.; Li, G. Anaerobic digestion of different agricultural wastes: A techno-economic assessment. *Bioresour. Technol.* **2020**, *315*, 123836.
(41) Zhang, C.; Xiao, G.; Peng, L.; Su, H.; Tan, T. The anaerobic co-digestion of food waste and cattle manure. *Bioresour. Technol.* **2013**, *129*, 170–176.
(42) Li, Y.; Zhang, R.; Liu, G.; Chen, C.; He, Y.; Liu, X. Comparison of methane production potential, biodegradability, and kinetics of different organic substrates. *Bioresour. Technol.* **2013**, *149*, 565–569.
(43) Triolo, J. M.; Sommer, S. G.; Møller, H. B.; Weisbjerg, M. R.; Jiang, X. Y. A new algorithm to characterize biodegradability of biomass during anaerobic digestion: influence of lignin concentration on methane production potential. *Bioresour. Technol.* **2011**, *102*, 9395–9402.
(44) Buffiere, P.; Loisel, D.; Bernet, N.; Delgenes, J. P. Towards new indicators for the prediction of solid waste anaerobic digestion properties. *Water Sci Technol* **2006**, *53*, 233–241.
(45) Møller, H. B.; Sommer, S. G.; Ahring, B. K. Methane productivity of manure, straw and solid fractions of manure. *Biomass and Bioenergy* **2004**, *26*, 485–495.
(46) Qin, X.; Zhong, W.; Wang, R.; Li, Z.; Gao, X.; Li, G. Assessment of food waste biodegradability by biochemical methane potential tests. *Energy Sources, Part A: Recovery, Utilization, and Environmental Effects* **2016**, *38*, 3599–3605.
(47) Zheng, W.; Phoungthong, K.; Lü, F.; Shao, L.-M.; He, P.-J. Evaluation of a classification method for biodegradable solid wastes using anaerobic degradation parameters. *Waste Manag.* **2013**, *33*, 2632–2640.
(48) Ward, A. J.; Hobbs, P. J.; Holliman, P. J.; Jones, D. L. Optimisation of the anaerobic

digestion of agricultural resources. *Bioresour. Technol.* **2008**, *99*, 7928–7940.
(49) Pabón Pereira, C. P.; Slingerland, M.; Van Lier, J. B.; Rudyrabbinge. Anaerobic digestion as a key technology for biomass valorization: contribution to the energy balance of biofuel chains. In *The Biogas Handbook*; Elsevier, 2013; pp. 166–188.
(50) Neshat, S. A.; Mohammadi, M.; Najafpour, G. D.; Lahijani, P. Anaerobic co-digestion of animal manures and lignocellulosic residues as a potent approach for sustainable biogas production. *Renewable and Sustainable Energy Reviews* **2017**, *79*, 308–322.
(51) Gunaseelan, V. N. Biochemical methane potential of fruits and vegetable solid waste feedstocks. *Biomass and Bioenergy* **2004**, *26*, 389–399.
(52) U.S. Bureau of Labor Statistics. Employment, hours, and earnings from the current employment statistics survey (Series ID: CEU3232500008). https://data.bls.gov/cgi-bin/srgate (accessed Aug 30, 2020).
(53) Davis, R.; Tao, L.; Tan, E. C.; Biddy, M. J.; Beckham, G. T.; Scarlata, C.; Jacobson, J.; Cafferty, K.; Ross, J.; Lukas, J.; et al. *Process design and economics for the conversion of lignocellulosic biomass to hydrocarbons: dilute-acid and enzymatic deconstruction of biomass to sugars and biological conversion of sugars to hydrocarbons* ; NREL/TP-5100-60223.; National Renewable Energy Lab. (NREL): Golden, CO (United States), 2013.
(54) Argonne National Laboratory. *The Greenhouse Gases, Regulated Emissions, and Energy Use in Technologies (GREET®) Model*; Argonne National Laboratory, 2021.
(55) Alhashimi, H. A.; Aktas, C. B. Life cycle environmental and economic performance of biochar compared with activated carbon: A meta-analysis. *Resources, Conservation and Recycling* **2017**, *118*, 13–26.
(56) Wernet, G.; Bauer, C.; Steubing, B.; Reinhard, J.; Moreno-Ruiz, E.; Weidema, B. The ecoinvent database version 3 (part I): overview and methodology. *Int. J. Life Cycle Assess.* **2016**, *21*, 1218–1230.
(57) NREL. Life Cycle Assessment Harmonization. https://www.nrel.gov/analysis/life-cycle-assessment.html (accessed Apr 6, 2021).
(58) Joshi, J.; Wang, J. Manure management coupled with bioenergy production: An environmental and economic assessment of large dairies in New Mexico. *Energy Economics* **2018**, *74*, 197–207.
(59) Zhang, Y.; White, M. A.; Colosi, L. M. Environmental and economic assessment of integrated systems for dairy manure treatment coupled with algae bioenergy production. *Bioresour. Technol.* **2013**, *130*, 486–494.
(60) Good, N. D. *Documentation for Greenhouse Gas Emission and Energy Factors Used in the Waste Reduction Model (WARM).*; epa.gov, 2020.
(61) Silva, C. B.; Valente, L. M. P.; Matos, E.; Brandão, M.; Neto, B. Life cycle assessment of aquafeed ingredients. *Int. J. Life Cycle Assess.* **2017**, *23*, 1–23.
(62) Bernard, J. K. Oilseed and oilseed meals. In *Reference module in food science*; Elsevier, 2016.
(63) Saha, B. C.; Yoshida, T.; Cotta, M. A.; Sonomoto, K. Hydrothermal pretreatment and enzymatic saccharification of corn stover for efficient ethanol production. *Ind. Crops Prod.* **2013**, *44*, 367–372.
(64) Lloyd, T. A.; Wyman, C. E. Combined sugar yields for dilute sulfuric acid pretreatment of corn stover followed by enzymatic hydrolysis of the remaining solids. *Bioresour. Technol.* **2005**, *96*, 1967–1977.

(65) Bolado-Rodríguez, S.; Toquero, C.; Martín-Juárez, J.; Travaini, R.; García-Encina, P. A. Effect of thermal, acid, alkaline and alkaline-peroxide pretreatments on the biochemical methane potential and kinetics of the anaerobic digestion of wheat straw and sugarcane bagasse. *Bioresour. Technol.* **2016**, *201*, 182–190.

(66) Sambusiti, C.; Monlau, F.; Ficara, E.; Carrère, H.; Malpei, F. A comparison of different pre-treatments to increase methane production from two agricultural substrates. *Appl. Energy* **2013**, *104*, 62–70.

(67) Song, Z.; GaiheYang; Liu, X.; Yan, Z.; Yuan, Y.; Liao, Y. Comparison of seven chemical pretreatments of corn straw for improving methane yield by anaerobic digestion. *PLoS One* **2014**, *9*, e93801.

(68) Zheng, M.; Li, X.; Li, L.; Yang, X.; He, Y. Enhancing anaerobic biogasification of corn stover through wet state NaOH pretreatment. *Bioresour. Technol.* **2009**, *100*, 5140–5145.

(69) Costa, A. G.; Pinheiro, G. C.; Pinheiro, F. G. C.; Dos Santos, A. B.; Santaella, S. T.; Leitão, R. C. The use of thermochemical pretreatments to improve the anaerobic biodegradability and biochemical methane potential of the sugarcane bagasse. *Chemical Engineering Journal* **2014**, *248*, 363–372.

(70) Pang, Y. Z.; Liu, Y. P.; Li, X. J.; Wang, K. S.; Yuan, H. R. Improving biodegradability and biogas production of corn stoverthrough sodium hydroxide solid state pretreatment. *Energy Fuels* **2008**, *22*, 2761–2766.

(71) Chandra, R.; Takeuchi, H.; Hasegawa, T.; Kumar, R. Improving biodegradability and biogas production of wheat straw substrates using sodium hydroxide and hydrothermal pretreatments. *Energy* **2012**, *43*, 273–282.

(72) Dussadee, N.; Ramaraj, R.; Cheunbarn, T. Biotechnological application of sustainable biogas production through dry anaerobic digestion of Napier grass. *3 Biotech* **2017**, *7*, 47.
